# Watchdog – a workflow management system for the distributed analysis of large-scale experimental data

**DOI:** 10.1186/s12859-018-2107-4

**Published:** 2018-03-13

**Authors:** Michael Kluge, Caroline C. Friedel

**Affiliations:** Institute for Informatics, Ludwig-Maximilians-Universität München, Amalienstraße 17, München, 80333 Germany

**Keywords:** Workflow management system, High-throughput experiments, Large-scale datasets, Automated execution, Distributed analysis, Reusability, Reproducibility, RNA-seq

## Abstract

**Background:**

The development of high-throughput experimental technologies, such as next-generation sequencing, have led to new challenges for handling, analyzing and integrating the resulting large and diverse datasets. Bioinformatical analysis of these data commonly requires a number of mutually dependent steps applied to numerous samples for multiple conditions and replicates. To support these analyses, a number of workflow management systems (WMSs) have been developed to allow automated execution of corresponding analysis workflows. Major advantages of WMSs are the easy reproducibility of results as well as the reusability of workflows or their components.

**Results:**

In this article, we present *Watchdog*, a WMS for the automated analysis of large-scale experimental data. Main features include straightforward processing of replicate data, support for distributed computer systems, customizable error detection and manual intervention into workflow execution. *Watchdog* is implemented in Java and thus platform-independent and allows easy sharing of workflows and corresponding program modules. It provides a graphical user interface (GUI) for workflow construction using pre-defined modules as well as a helper script for creating new module definitions. Execution of workflows is possible using either the GUI or a command-line interface and a web-interface is provided for monitoring the execution status and intervening in case of errors. To illustrate its potentials on a real-life example, a comprehensive workflow and modules for the analysis of RNA-seq experiments were implemented and are provided with the software in addition to simple test examples.

**Conclusions:**

*Watchdog* is a powerful and flexible WMS for the analysis of large-scale high-throughput experiments. We believe it will greatly benefit both users with and without programming skills who want to develop and apply bioinformatical workflows with reasonable overhead. The software, example workflows and a comprehensive documentation are freely available at www.bio.ifi.lmu.de/watchdog.

**Electronic supplementary material:**

The online version of this article (10.1186/s12859-018-2107-4) contains supplementary material, which is available to authorized users.

## Background

The development of high-throughput experimental methods, in particular next-generation-sequencing (NGS), now allows large-scale measurements of thousands of properties of biological systems in parallel. For example, modern sequencing platforms now allow simultaneously quantifying the expression of all human protein-coding genes and non-coding RNAs (RNA-seq [1]), active translation of genes (ribosome profiling [2]), transcription factor binding (ChIP-seq [3]), and many more. Dissemination of these technologies combined with decreasing costs resulted in an explosion of large-scale datasets available. For instance, the ENCODE project, an international collaboration that aims to build a comprehensive list of all functional elements in the human genome, currently provides data obtained in more than 7000 experiments with 39 different experimental methods [4]. While such large and diverse datasets still remain the exception, scientific studies now commonly combine two or more high-throughput techniques for several conditions or in time-courses in multiple replicates (e.g. [5–7]).

Analysis of such multi-omics datasets is quite complex and requires a lot of mutually dependent steps. As a consequence, large parts of the analysis often have to be repeated due to modifications of initial analysis steps. Furthermore, errors e.g. due to aborted program runs or improperly set parameters at intermediate steps have consequences for all downstream analyses and thus have to be monitored. Since each analysis consists of a set of smaller tasks (e.g read quality control, mapping against the genome, counting of reads for gene features), it can usually be represented in a structured way as a workflow. Automated execution of such workflows is made possible by workflow management systems (WMSs), which have a number of advantages.

First, a workflow documents the steps performed during the analysis and ensures reproducibility. Second, the analysis can be executed in an unsupervised and parallelized manner for different conditions and replicates. Third, workflows may be reused for similar studies or shared between scientists. Finally, depending on the specific WMS, users with limited programming skills or experience with the particular analysis tools applied within the workflow may more or less easily apply complicated analyses on their own data. On the downside, the use of a WMS usually requires some initial training and some overhead for the definition of workflows. Moreover, the WMS implementation itself might restrict which analyses can be implemented as workflows in the system. Nevertheless, the advantages of WMSs generally outweigh the disadvantages for larger analyses.

In recent years, several WMS have been developed that address different target groups or fields of research or differ in the implemented set of features. The most well-known example, *Galaxy*, was initially developed to enable experimentalists without programming experience to perform genomic data analyses in the web browser [8]. Other commonly used WMSs are *KNIME* [9], an open-source data analysis platform which allows programmers to extend its basic functionality by adding new Java programs, and *Snakemake* [10], a python-based WMS. *Snakemake* allows definition of tasks based on rules and automatically infers dependencies between tasks by matching filenames. A more detailed comparison of these WMSs is given in the Results and discussion section.

In this article, we present *Watchdog*, a WMS designed to support bioinformaticians in the analysis of large high-throughput datasets with several conditions and replicates. *Watchdog* offers straightforward processing of replicate data and easy outsourcing of resource-intensive tasks on distributed computer systems. Additionally, *Watchdog* provides a sophisticated error detection system that can be customized by the user and allows manual intervention. Individual analysis tasks are encapsulated within so-called modules that can be easily shared between developers. Although *Watchdog* is implemented in Java, there is no restriction on which programs can be included as modules. In principle, *Watchdog* can be deployed on any operating system.

Furthermore, to reduce the overhead for workflow design, a GUI is provided, which also enables users without programming experience to construct and run workflows using pre-defined modules. As a case study on how *Watchdog* can be applied, modules for read quality checks, read mapping, gene expression quantification and differential gene expression analysis were implemented and a workflow for analyzing differential gene expression in RNA-seq data was created. *Watchdog*, including documentation, implemented modules as well as the RNA-seq analysis workflow and smaller test workflows can be obtained at www.bio.ifi.lmu.de/watchdog.

## Implementation

### Overview of *Watchdog*

**Fig. 1:**
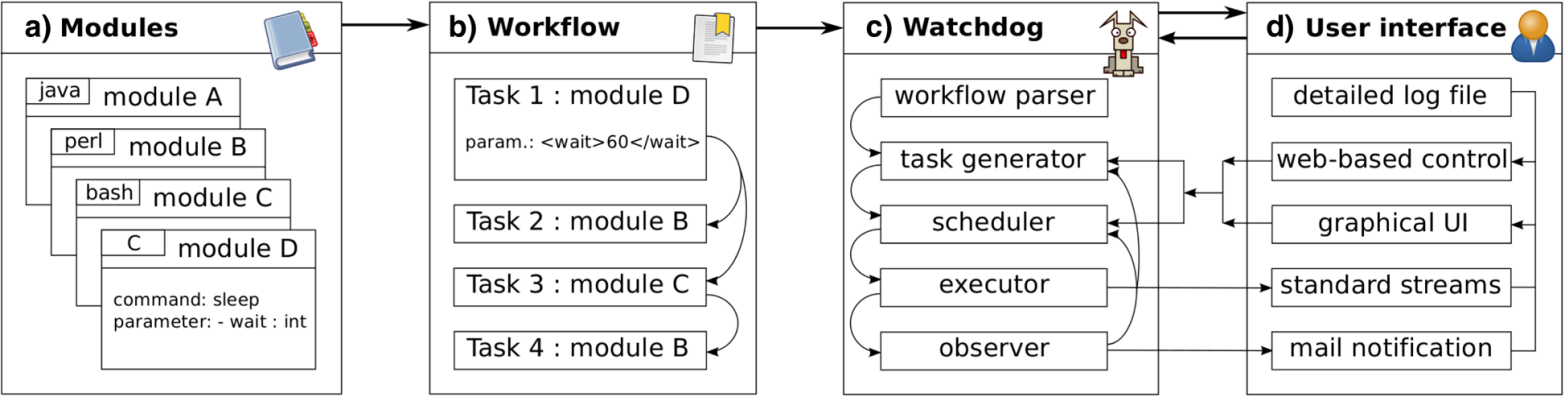
Overview of *Watchdog*. **a** Modules are defined in an XSD format that describes the command to be executed and valid parameters. All modules together represent the software library that can be used in workflows and can be extended by defining new modules. **b** A workflow is defined in an XML format and consists of tasks that depend on each other. Among others, the XML format allows setting environment variables, defining different executors in the *settings* part of the workflow and processing replicate data in a straightforward way. **c***Watchdog* parses the workflow, creates the corresponding tasks, executes them and verifies whether execution of each task terminated successfully or not. **d** Email notification (optional) and log files combined with either the GUI or a simple web-interface allow monitoring the execution of the workflow and intervening if necessary, e.g. by restarting tasks with modified parameters

The core features of *Watchdog* and their relationships are outlined in Fig. 1 and briefly described in the following. More details and additional features not mentioned in this overview are described in subsequent sections, Additional files 1, 2 and 3 and in the manual available at www.bio.ifi.lmu.de/watchdog.

#### Modules

Modules encapsulate re-usable components that perform individual tasks, e.g. mapping of RNA-seq data, counting reads for gene features or visualizing results of downstream analyses. Each module is declared in an XSD file containing the command to execute and the names and valid ranges of parameters. In addition to the XSD file, a module can contain scripts or compiled binaries required by the module and a test script running on example data. Module developers are completely flexible in the implementation of individual modules. They can use the programming language of their choice, include binaries with their modules or automatically deploy required software using Conda (https://conda.io/), Docker (https://www.docker.com/, an example module using a Docker image for Bowtie 2 [11] is included with *Watchdog*) or similar tools. Furthermore, *Watchdog* provides a helper bash script to generate the XSD definition file for new modules and (if required) a skeleton bash script that only needs to be extended by the program call.

Essentially, any program that can be run from the command-line can be used in a module and several program calls can be combined in the same module using e.g. an additional bash script. In principle, a module could even contain a whole pipeline, such as Maker-P [12], but this would run counter the purpose of a WMS. Here, it would make more sense to separate the individual steps of the pipeline into different modules and then implement the pipeline as a *Watchdog* workflow. Finally, *Watchdog* is not limited to bioinformatics analyses, but can be also used for workflows from other domains.

#### Workflows

**Fig. 2:**
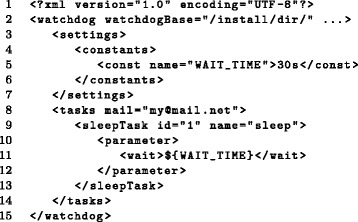
Simple workflow in XML format. This example shows a simple *Watchdog* workflow executing a 30 second sleep task. A constant named *WAIT_TIME* is defined within the *settings* environment (line 5). Email notification of the user is enabled using the optional *mail* attribute of the *tasks* environment (line 8). Here, a task of type *sleepTask* with *id* 1 and *name* sleep is defined (lines 9-13). Either id or name can be used to refer to this task in dependency declarations of other tasks. Within the *parameter* environment of the *sleepTask*, values are assigned to required parameters (lines 10-12), which were specified in the XSD file of this particular module. In this case, the parameter *wait* is set to the value stored in the constant *WAIT_TIME* (line 11)

Workflows are defined in XML and specify a sequence of tasks to be executed, the values of their input parameters and dependencies between them. An example for a simple workflow is given in Fig. 2. Among other features that are described later, it is possible to define constants, environment variables and execution hosts in a dedicated *settings* element at the beginning of the workflow, redirect the standard error and standard output for individual tasks or define how detailed the user is informed on the execution status of tasks.

The advantage of XML is that it is widely used in many contexts. Thus, a large fraction of potential *Watchdog* users should already be familiar with its syntax and only need to learn the *Watchdog* XML schema. Furthermore, numerous XML editors are available, including plugins for the widely used integrated development environment (IDE) *Eclipse* [13], which allow XML syntax checking and document structure highlighting. Finally, a number of software libraries for programmatically loading or writing XML are also available (e.g. Xerces for Java, C++ and Perl (http://xerces.apache.org/), ElementTree in Python).

In addition, *Watchdog* also provides an intuitive GUI (denoted *workflow designer*) that can be used to design a workflow, export the corresponding XML file afterwards and run the workflow in the GUI.

#### *Watchdog*

The core element of *Watchdog* that executes the workflow was implemented in Java and therefore is, in principle, platform-independent. Individual modules, however, may depend on the particular platform used. For instance, if a module uses programs only available for particular operating systems (e.g. Linux, macOS, Windows), it can only be used for this particular system.

As a first step, *Watchdog* validates the XML format of the input workflow and parses the XML file. Based on the XML file, an initial set of dependency-free tasks, i.e. tasks that do not depend on any other tasks, is generated and added to the WMS scheduler to execute them. Subsequently, the scheduler continuously identifies tasks for which dependencies have been resolved, i.e. all preceding tasks the task depends on have been executed successfully, and schedules them for execution. Once a task is completed, *Watchdog* verifies that the task finished successfully. In this case, the task generator and scheduler are informed since dependencies of other tasks might have become resolved. In case of an error, the user is informed via email (optional) and the task is added to the scheduler again but is blocked for execution until the user releases the block or modifies its parameters. Alternatively, the user may decide to skip the task or mark the error as resolved.

#### User interfaces

**Fig. 3:**
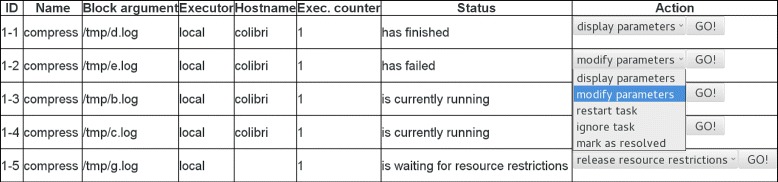
Web-interface of *Watchdog*. Each line of the table provides information on the status of a task or subtask. The drop-down menu at the end of each line allows to perform specific actions depending on the status of the task. The menu is shown for subtask *1-2*, which could not be executed successfully. To generate this screenshot the example workflow depicted in Fig. 6 was processed, which compresses all log-files stored in directory */tmp/*. Since the number of simultaneously running subtasks was set to at most 2 for this task, subtask *1-5* is put on hold until subtasks *1-3* and *1-4* have finished or the user manually releases the resource restriction

*Watchdog* provides both a command-line version as well as a GUI that can be used to execute workflows and to keep track of their processing. Moreover, a web-interface is provided to GUI and command-line users that displays the status of all tasks in a table-based form and allows monitoring and interacting with the execution of tasks by releasing scheduled tasks, changing parameters after a failed task execution and more (see Fig. 3). The link to the web-interface is either printed to standard output or sent to the user by email if they enabled email notification. In the latter case, the user will also be notified per email about execution failure (always) or success (optional). Finally, the command-line interface also allows resuming a workflow at any task or limiting the execution of the workflow to a subset of tasks using the -start (start execution at specified task), -stop (stop execution after specified task), -include (include this task in execution) and -exclude (exclude this task for execution) options.

In the following more details are provided on principles and possibilities of workflow design in *Watchdog* and defining custom modules. The GUI is described in detail in Additional file 1.

### Process blocks for creating subtasks

Analysis of high-throughput data often requires performing the same analysis steps in parallel for a number of samples representing different conditions or biological or technical replicates. To support these types of analyses, *Watchdog* uses so-called process blocks to automatically process tasks that differ only in values of parameters, e.g. short read alignment for all FASTQ files in a directory. For this purpose, process blocks define a set of instances, each of which contain one or more variables. For each instance, one subtask is created and subtask placeholders in the task definition are replaced with the variable values of the instance. For the example in which a task is executed for all FASTQ-files in a directory, each instance holds one variable containing the absolute file path of the file. The number of subtasks corresponds to the number of FASTQ-files in the directory.

**Fig. 4:**
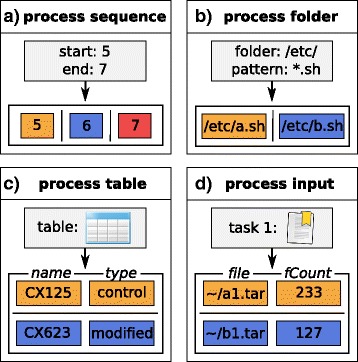
Types of process blocks. With the help of process blocks, multiple tasks that differ only in the parameter values can be created without defining all of them separately. Four different types of process blocks are implemented that fall into two general classes. Instances of the first class contain only a single variable, either (**a**) a value from a numerical sequence (process sequence) or (**b**) a path to files (process folder). In (**a**), subtasks are created based on an integer sequence starting at 5 and ending at 7 with an increment of 1. In (**b**), a subtask is created for each sh-file in the folder */etc/*. Instances of the second type can contain multiple variables, either (**c**) instances derived from tables (process table) or (**d**) instances based on return values returned by previous tasks this task depends on (process input). In (**c**), a table with two columns named *name* and *type* and two rows is used as input for the process table. This results in two subtasks for this task, one for each row. The process input block in (**d**) depends on a task with id 1, which itself had two subtasks. Hence, this task returns two instances, each containing the variables *file* and *fCount* obtained from its return variables

Currently four different types of process blocks are supported by *Watchdog*: process sequences, process folders, process tables and process input (Fig. 4). In case of process sequences (Fig. 4a) and process folders (Fig. 4b), instances only hold a single variable. Process sequences are comparable to for-loops as they generate instances containing numerical values (integer or floating-point numbers) with a fixed difference between two consecutive numbers (default: 1). Instances generated by process folders contain the absolute path to files and are generated based on a parent folder and a filename pattern.

Process tables (Fig. 4c) and process input (Fig. 4d) blocks can generate instances with multiple variables. Instances generated by a process table are based on the content of a tab-separated file. The rows of the table define individual instances and the columns the variables for each instance. In case of process input blocks, variables and instances are derived from return values of preceding tasks the task depends on.

**Fig. 5:**
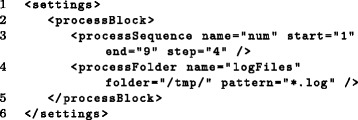
Definition of process blocks. In this example, two process blocks are defined within the *processBlock* environment (lines 2-5). In line 3, a process sequence named *num* is defined consisting of three instances (1, 5 and 9). In line 4, a process folder selecting all log-files in the */tmp/* directory is defined

**Fig. 6:**
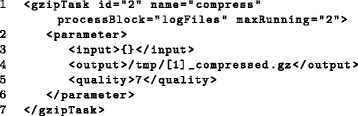
Usage of process blocks. The process block *logFiles* defined in Fig. 5 is used to generate several subtasks (line 1). These subtasks create compressed versions of the log-files stored in */tmp/*. In this case, at most two subtasks are allowed to run simultaneously. Additional file 2 describes how process block variables can be accessed. Here, the placeholder {} is replaced by the variable values stored in the process block, i.e. the complete file paths, and [1] is replaced with the file names (without the ‘.log’ file-ending) (lines 3-4)

Figure 5 shows an example how process blocks can be defined and Fig. 6 shows how they can be used for creation of subtasks. In Additional file 2, a detailed description with examples is provided on how to use process blocks for the analysis of data sets with several replicates or conditions. Furthermore, *Watchdog* provides a plugin system that allows users with programming skills to implement novel types of process blocks without having to change the original *Watchdog* code (see Additional file 3).

### Dependencies

**Fig. 7:**
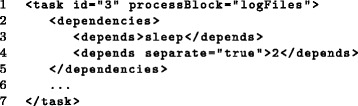
Definition of dependencies. The task defined in this example creates subtasks using the process block *logFiles* from Fig. 5 (line 1) with both task and subtask dependencies. A task dependency on the task *sleep* defined in Fig. 2 is indicated in line 3. In addition, subtask dependencies to the task with id 2 defined in Fig. 6 are indicated in line 4. In this case, each subtask depends on the subtask of task 2 which was created using the same instance defined by the process block *logFiles*, i.e. the same file path

By default, all tasks specified in a *Watchdog* workflow are independent of each other and are executed in a non-deterministic order. Alternatively, dependencies on either task or subtask level (details in the next paragraphs) can be defined using the *id* or *name* attribute of a task (see Fig. 7). Dependency definitions impose a partial order on tasks, meaning that tasks depending on other tasks will only be executed after those other tasks have finished successfully. Tasks without dependencies or resolved dependencies will still be executed in a non-deterministic order.

Although explicit dependency definition adds a small manual overhead compared to automatic identification based on in- and output filenames as in *Snakemake*, it also provides more flexibility as dependencies can be defined that are not obvious from filenames. For instance, analysis of sequencing data usually involves quality control of sequencing reads, e.g. with FastQC [14], before mapping of reads, and users might want to investigate the results of quality control before proceeding to read mapping. However, output files of quality control are not an input to read mapping and thus this dependency could not be identified automatically. To provide more time to manually validate results of some intermediate steps, *Watchdog* allows adding checkpoints after individual tasks. After completion of a task with checkpoint, all dependent tasks are put on hold until the checkpoint is released. All checkpoints in a workflow can be deactivated upon workflow execution with the -disableCheckpoint flag of the *Watchdog* command-line version.

#### Task dependencies

**Fig. 8:**
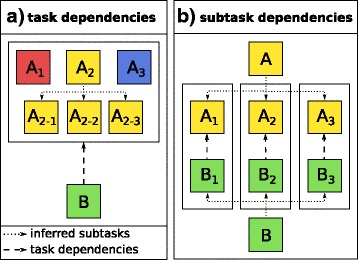
Types of dependencies. Dependencies can either be defined on (**a**) task or (**b**) subtask level. **a** Task *B* depends on tasks *A*_1_, *A*_2_ and *A*_3_. Task *A*_2_ uses a process block to create the three subtasks *A *_2−1_, *A *_2−2_ and *A *_2−3_. Task *B* will be executed when *A*_1_, *A*_2_ (including all subtasks) and *A*_3_ have finished successfully. **b** Tasks *A* and *B* create subtasks using a process block. For example, task *A* might decompress files stored in a folder (by using a process folder) and task *B* might extract data from the decompressed files afterwards (by using a process input block). Here, subtask *B *_*x*_ of *B* only depends on the subtask *A *_*x*_ of *A* based on whose return values it is created

A task *B* can depend on one or more other tasks *A*_1_ to *A*_*n*_, which means that execution of task *B* is put on hold until tasks *A*_1_ to *A*_*n*_ have finished successfully. If some of the dependencies *A*_1_ to *A*_*n*_ use process blocks to create subtasks, task *B* is put on hold until all subtasks are finished successfully. Figure 8a illustrates the described behavior on a small example in which task *B* depends on three other tasks.

#### Subtask dependencies

If a subtask *B*_*x*_ of a task *B* only depends on a particular subtask *A*_*x*_ of *A* instead of all subtasks of *A*, the definition of subtask dependencies in the workflow allows executing *B*_*x*_ as soon as *A*_*x*_ has finished successfully (but not necessarily other subtasks of *A*). This is illustrated in Fig. 8b and can be explained easily for the most simple case when the process block used for task *B* is a process input block containing the return values of subtasks of *A*. In that case, a subtask *B*_*x*_ depends only on the subtask *A*_*x*_ of *A* that returned the instance resulting in the creation of *B*_*x*_. The use of subtask dependencies is particularly helpful if subtasks of *A* need different amounts of time to finish or cannot all be executed at the same time due to resource restrictions, such as a limited amount of CPUs or memory available. In this case, *B*_*x*_ can be executed as soon as *A*_*x*_ has finished but before all other subtasks of *A* have finished. An example application would be the conversion of SAM files resulting from read mapping (task *A*) to BAM files (task *B*).

### Parallel and distributed task execution

**Fig. 9:**
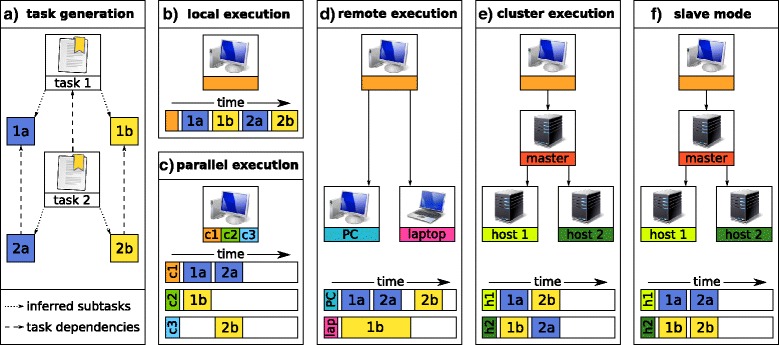
Parallel and distributed task execution. Three different types of executors are implemented in *Watchdog*: (i) execution on the local host that runs *Watchdog*, (ii) remote execution via SSH or (iii) cluster execution using DRMAA or the Slurm Workload Manager. **a** In this example, the four subtasks 1a, 1b, 2a and 2b are created by *Watchdog* based on tasks 1 and 2 using process blocks. Task 2a depends on 1a, and 2b on 1b. All tasks are assumed to require the same runtime. **b** By default, one task is executed after the other on the host running *Watchdog*. **c***Watchdog* also allows parallel execution in all three execution modes (local, remote and cluster execution). **d** For remote execution, *Watchdog* establishes a SSH connection to pre-defined execution hosts and randomly distributes the tasks that should be executed to these execution hosts. **e** For cluster execution, the DRMAA or Slurm master receives tasks to execute and redirects them to its execution hosts. *Watchdog* has no influence on which execution host is used for task execution because the tasks are distributed by the internal DRMAA or Slurm scheduler. **f** During slave mode (supported for remote and cluster execution), tasks or subtasks that depend on each other are scheduled on the same execution host, which allows using the local disk space of the host for storage of files that are needed only temporarily but by different tasks

By default all tasks are executed one after the other on the host running *Watchdog* (see Fig. 9a,b). In principle, however, tasks that are independent of each other or individual subtasks of a task can be executed in parallel. *Watchdog* implements three different types of executors that facilitate parallel execution of tasks: (i) local executor (Fig. 9c), (ii) remote executor (Fig. 9d) and (iii) cluster executor (Fig. 9e). All executors allow multi-threaded execution of tasks. In cases (i) and (ii) *Watchdog* uses multiple threads for parallel execution of tasks while in case (iii) the cluster master is utilized to distribute tasks on the cluster. Before execution or after completion or failure of tasks, files or directories can be created, deleted or copied to/from remote file systems (e.g. the file system of a remote or cluster executor) using so-called task actions. By default, *Watchdog* supports virtual file systems based on the protocols File, HTTP, HTTPS, FTP, FTPS and SFTP as well as the main memory (RAM). However, any file system with an implementation of the FileProvider interface from the Commons Virtual File System project of the Apache Software Foundation (http://commons.apache.org/proper/commons-vfs/) can also be used (see manual).

**Fig. 10:**
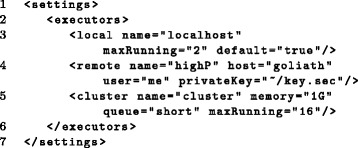
Defining executors. This example defines three possible executors: (i) the local host running *Watchdog* using two parallel threads for task execution (line 3). This will be used by default for task execution if no other executor is specified in a task definition using the *executor* attribute. (ii) a remote host named *goliath* accessed by SSH and authenticated via a private key that should be protected by a passphrase (line 4). (iii) a cluster executor that schedules a maximum of 16 simultaneously running tasks on the *short* queue of a computer cluster supporting DRMAA (line 5)

Executors and their resource limitations are declared in the *settings* element at the beginning of the workflow (see Fig. 10) and assigned to tasks based on their names. Within each workflow, an arbitrary number of executors of different types can be defined and any of these can be assigned to individual tasks. For instance, memory-intensive tasks might be executed on a dedicated high-memory computer using a remote executor while other tasks spawning many subtasks are distributed using a cluster executor and non-resource-intensive tasks are run using a local executor. Here, the number of simultaneously running (sub)tasks can be restricted on task (see Fig. 6) or executor level (see Fig. 10), e.g. to not occupy the whole cluster with many long-running tasks. Provided the name of a particular executor remains the same, everything else can be modified about this executor without having to change the *tasks* part of the workflow. This includes not only resource limitations or the maximum number of running tasks but even the type of executor, for instance when moving the workflow to a different system.

Every host that accepts secure shell connections (SSH) can be used as a remote executor (see Fig. 9d). In this case, a passphrase-protected private key for user authentication must be provided. For cluster execution, any grid computing infrastructures that implement the Distributed Resource Management Application API (DRMAA) can be utilized (see Fig. 9e). By default, *Watchdog* uses the Sun Grid Engine (SGE) but other systems that provide a DRMAA Java binding can also be used. Furthermore, *Watchdog* provides a plugin system that allows users with programming skills to add new executor types without having to change the original *Watchdog* code. This plugin system is explained in detail in Additional file 3 and was used to additionally implement an executor for computing clusters or supercomputers running the Slurm Workload Manager (https://slurm.schedmd.com/). The plugin system can also be used to provide support for cloud computing services that do not allow SSH. Support for the Message Passing Interface (MPI) is not explicitly modeled in *Watchdog*, but MPI can be used by individual modules if it is supported by the selected executor.

Finally, to allow storage of potentially large temporary files on the local hard disk of cluster execution hosts and sharing of these files between tasks, *Watchdog* also implements a so-called *slave mode* (see Fig. 9f). In slave mode, the scheduler ensures that tasks or subtasks depending on each other are processed on the same host allowing them to share temporary files on the local file system. For this purpose, a new slave is first started on an execution host, which establishes a network connection to the master (i.e. the host running *Watchdog*) and then receives tasks from the master for processing.

### Error detection and handling

**Fig. 11:**
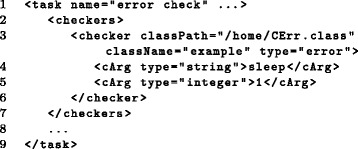
Invocation of a custom error checker. The example illustrates how a custom error checker implemented in class *CErr* located in directory */home/* can be added to a task (line 3). In line 4 and 5, two arguments of type *string* and *integer* are forwarded to the constructor of the error checker

During execution of workflows, a number of errors can occur resulting either in aborted program runs or incorrect output. To identify such errors, *Watchdog* implements a sophisticated error checking system that allows flexible extension by the user. For this purpose, *Watchdog* first checks the exit code of the executed module. By definition an exit code of zero indicates that the called command was executed successfully. However, some tools return zero as exit code regardless of whether the command succeeded or failed. Thus, the exit code alone is not a reliable indicator whether the command was executed successfully. Furthermore, a command can technically succeed without the desired result being obtained. For instance, the mapping rate for RNA-seq data may be very low due to wrong parameter choices or low quality of reads. To handle such cases, the user has the option to implement custom success and error checkers in Java that are executed by *Watchdog* after a task is finished. Two steps must be performed to use custom checkers: implementation in Java and invocation in the XML workflow (see Fig. 11 for an example and the manual for details).

Once the task is finished, the checkers are evaluated in the same order as they were added in the XML workflow. In cases in which both success and error were detected by different checkers, the task will be treated as failed. When an error is detected, the user is informed via email notification (if enabled, otherwise the information is printed to standard output), including the name of the execution host, the executed command, the returned exit code and the detected errors.

Information on failure or success is also available via the web-interface, which then allows to perform several actions: (i) modify the parameter values for the task and restart it, (ii) simply restart the task, (iii) ignore the failure of the task or (iv) manually mark the task as successfully resolved. In case of (iii), (sub)tasks that depend on that task will not be executed, but other (sub)tasks will continue to be scheduled and executed. To continue with the processing of tasks depending on the failed task, option (iv) can be used. In this case, values of return parameters of the failed task can be entered manually via the web-interface.

Option (i) is useful if a task was executed with inappropriate parameter values and avoids having to restart the workflow at this point and potentially repeating tasks that are defined later in the workflow but are not dependent on the failed task. As *Watchdog* aims to execute all tasks without (unresolved) dependencies as soon as executors and resource limitations allow, these other tasks might already be running or even be finished. Option (ii) is helpful if a (sub)task fails due to some temporary technical problem in the system, a bug in a program used in the corresponding module or missing software. The user can then restart the (sub)task as soon as the technical problem or the bug is resolved or the software has been installed without having to restart the other successfully finished or still running (sub)tasks. Here, the XSD definition of a module cannot be changed during a workflow run as XSD files are loaded at the beginning of workflow execution, but the underlying program itself can be modified as long as the way it is called remains the same. Option (iii) allows to finish an analysis for most samples of a larger set even if individual samples could not be successfully processed, e.g. due to corrupt data. Finally, option (iv) is useful if custom error checkers detect a problem with the results, but the user nevertheless wants to finish the analysis.

### Defining custom modules

*Watchdog* is shipped with 20 predefined modules, but the central idea of the module concept is that every developer can define their own modules, use them in connection with *Watchdog* or share them with other users. Each module consists of a folder containing the XSD module definition file and optional scripts, binaries and test scripts. It should be noted here that while the complete encapsulation of tasks within modules is advantageous for larger tasks consisting of several steps or including additional checks on in- or output, the required module creation adds some burden if only a quick command is to be executed, such as a file conversion or creation of a simple plot. However, to reduce the resulting overhead for module creation, a helper bash script is available for unix-based systems that interactively leads the developer through the creation of the XSD definition file.

For this purpose, the script asks which parameters and flags to add. In addition, optional return parameters can be specified that are required if the module should be used as process input block. If the command should not be called directly because additional functions (e.g. checks for existence of input and output files and availability of programs) should be executed before or after the invocation of the command, the helper script can generate a skeleton bash script that has to be only edited by the developer to include the program and additional function calls. Please note that modules shipped with *Watchdog* were created with the helper script, thus XSD files and large fractions of bash scripts were created automatically with relatively little manual overhead. Once the XSD file for a module is created, the module can be used in a workflow. By default, *Watchdog* assumes that modules are located in a directory named *modules/* in the installation directory of *Watchdog*. However, the user can define additional module folders at the beginning of the workflow.

## Results and discussion

### Example workflows

For testing and getting to know the potentials of *Watchdog* by first-time users, two longer example workflows are provided with the software, which are documented extensively within the XML file (contained in the *examples* sub-directory of the *Watchdog* installation directory after configuring the examples, see manual for details). All example workflows can also be loaded into the GUI in order to get familiar with its usage (see Additional file 1). In order to provide workflows that can be used for practically relevant problems, 20 modules were developed that are shipped together with *Watchdog*. In addition, several smaller example workflows are provided, each demonstrating one particular feature of *Watchdog*. They are explained in detail in the manual. The next paragraphs describe the two longer example workflows and the corresponding test dataset.

#### Test dataset

A small test dataset consisting of RNA-seq reads is included in the *Watchdogexamples* directory. It is a subset of a recently published time-series dataset on HSV-1 lytic infection of a human cell line [5]. For this purpose, reads mapping to chromosome 21 were extracted for both an uninfected sample and a sample obtained after eight hours of infection. Both samples in total contain about 308,000 reads.

#### Workflow 1 - Basic information extraction

This workflow represents a simple example for testing *Watchdog* and uses modules encapsulating the programs *gzip*, *grep* and *join*, which are usually installed on unix-based systems by default. Processing of the workflow requires about 50MB of storage and less than one minute on a modern desktop computer. As a first step, gzipped FASTQ files are decompressed. Afterwards, read headers and read sequences are extracted into separate files. To demonstrate the ability of *Watchdog* to restrict the number of simultaneously running jobs, the sequence extraction tasks are limited to one simultaneous run, while the header extraction tasks are run in parallel (at most 4 simultaneously). Once the extraction tasks are finished, the resulting files from each sample are compressed and merged.

#### Workflow 2 - Differential gene expression

This workflow illustrates *Watchdog*’s potentials for running a more complex and practically relevant analysis. It implements a workflow for differential gene expression analysis of RNA-seq data and uses a number of external software programs for this purpose. Thus, although XSD files for corresponding modules are provided by *Watchdog*, the underlying software tools have to be installed and paths to binaries added to the environment before running this workflow. The individual modules contain dependency checks for the required software that will trigger an error if some of them are missing.

Software required by modules used in the workflow include *FastQC* [14], *ContextMap 2* [15], *BWA* [16], *samtools* [17], *featureCounts* [18], *RSeQC* [19], *R* [20], *DEseq* [21], *DEseq2* [22], *limma* [23], and *edgeR* [24]. The workflow can be restricted to just the initial analysis steps using the -start and -stop options of the *Watchdog* command-line version and individual analyses steps can be in- or excluded using the -include and -exclude options. Thus, parts of this workflow can be tested without having to install all programs. Please also note that the workflow was tested on Linux and may not immediately work on macOS due to differences in pre-installed software. Before executing the workflow a few constants have to be set, which are marked as *TODO* in the comments of the XML file. Processing of the workflow requires about 300MB of storage and a few minutes on a modern desktop computer.

The first step is again decompression of gzipped FASTQ files. Afterwards, quality assessment is performed for each replicate using *FastQC*, which generates various quality reports for raw sequencing data. Subsequently, the reads are mapped to chromosome 21 of the human genome using *ContextMap 2*. After read mapping is completed, the resulting SAM files are converted to BAM files and BAM files are indexed using modules based on *samtools*. Afterwards, reads are summarized to read counts per gene using *featureCounts*. As methods for differential gene expression detection may require replicates, pseudo-replicates are generated by running *featureCounts* twice with different parameters. This was done in order to provide a simple example that can be executed as fast as possible and should not be applied when real data is analyzed. In parallel, quality reports on the read mapping results are generated using *RSeQC*. Finally, *limma*, *edgeR*, *DEseq* and *DEseq2* are applied on the gene count table in order to detect differentially expressed genes. All four programs are run as part of one module, *DETest*, which also combines result tables of the different methods. Several of the provided modules also generate figures using *R*.

### Comparison with other WMSs

Most WMSs can be grouped into two types based on how much programming skills are required in order to create a workflow. If a well-engineered GUI or web interface is provided, users with basic computer skills should be able to create their own workflows. However, GUIs can also restrict the user as some features may not be accessible. Hence, a second group of WMSs addresses users with more advanced programming skills and knowledge of WMS-specific programming or scripting languages.

**Fig. 12:**
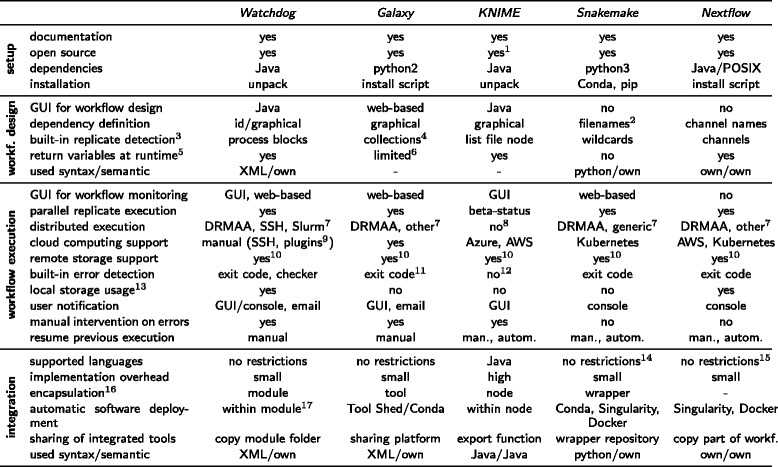
Comparison of *Watchdog* with other WMSs. Comparison was performed using features grouped into the categories setup, workflow design, workflow execution and integration. Workflow is abbreviated as *workf.* in this table. Integration refers to the integration of new data analysis tools into the particular WMS. Footnotes: ^1^six non-free extensions are available; ^2^since version 2.4.8, rules can also explicitly refer to the output of other rules; ^3^explanation: includes a way to automatically run a predefined workflow for a variable number of replicates based on filename patterns; ^4^have to be created manually in the web-interface from uploaded files; ^5^explanation: finished steps of the workflow can return variables that are used by subsequent steps as input; ^6^can only return the names of output files; ^7^other supported executors: *Watchdog*: new executors can be added with the plugin system, *Galaxy*: PBS/Torque, Open Grid Engine, Univa Grid Engine, Platform LSF, HTCondor, Slurm, Galaxy Pulsar, *Snakemake*: can also use cluster engines with access to a common file system and a submit command that accepts shell scripts as first argument, *Nextflow*: SGE, LSF, Slurm, PBS/Torque, NQSII, HTCondor, Ignite; ^8^non-free extensions for SGE or dedicated server support are available; ^9^custom executors for cloud computing services can be created using the plugin system; ^10^*Watchdog*: HTTP/S, FTP/S and SFTP by default, can be extended to any remote file system with an implementation of the FileProvider interface from the Commons Virtual File System project, *Galaxy*: Object Store plugins for S3, Azure, iRODS, *Snakemake*: S3, GS, SFTP, HTTP, FTP, Dropbox, XRootD, NCBI, WebDAV, GFAL, GridFTP. *Nextflow*: HTTP/S, FTP, S3; ^11^a hard-coded error checker triggered on keywords ‘exception’ and ‘error’ in standard output and error is provided; ^12^depends on the node implementation and left to developer; ^13^explanation: usage of local storage during distributed execution in order to avoid unnecessary load on the shared storage system; ^14^direct integration of python code is possible; ^15^own scripting language available; ^16^explanation: describes the concept used to separate workflow definition and functionality (e.g. *Watchdog*’s modules) in order to allow easy re-use of functionality; ^17^modules can include binaries in the module directory or automatically deploy required software using Conda, Singularity, Docker or similar tools available on the used system

As a comprehensive comparison of all available WMS is outside the scope of this article, two commonly used representatives of each group were selected and compared with *Watchdog*. Figure 12 lists features of each WMS, which are grouped into the categories *setup*, *workflow design*, *workflow execution* and *integration* of new tools. As representative WMSs *Galaxy* [8], *KNIME* [9], *Snakemake* [10], and *Nextflow* [25] were chosen. In the following paragraphs, the selected WMSs are discussed. Because all four WMSs as well as *Watchdog* allow non-programmers to execute predefined workflows, this property is not further discussed. Furthermore, an analysis of the computational overhead of *Watchdog* and *Snakemake* showed that the computational overhead of using either WMS (and likely any other) is negligible compared to the actual runtime of the executed tasks (see Additional file 4).

#### Galaxy

The most well-known WMS for bioinformatic analyses is *Galaxy* [8]. It was initially developed to enable experimentalists without programming experience to perform genomic data analyses in the web browser. Users can upload their own data to a *Galaxy* server, select and combine available analysis tools from a menu and configure them using web forms. To automatically perform the same workflow on several samples in a larger data set, so-called collections can be used.

In addition to computer resources, *Galaxy* provides a web-platform for sharing tools, datasets and complete workflows. Moreover, users can set up private *Galaxy* servers. In order to integrate a new tool, an XML-file has to be created that specifies the input and output parameters. Optionally, test cases and the expected output of a test case can be defined. Once the XML-file has been prepared, *Galaxy* must be made aware of the new tool and be re-started. If public *Galaxy* servers should be used, all input data must be uploaded to the public *Galaxy* servers. This is especially problematic for users with only low-bandwidth internet access who want to analyze large high-throughput datasets but cannot set up their own server.

In summary, *Galaxy* is a good choice for users with little programming experience who want to analyze data using a comfortable GUI, might not have access to enough computer resources for analysis of large high-throughput data otherwise, appreciate the availability of a lot of predefined tools and workflows and do not mind the manual overhead.

#### KNIME

The Konstanz Information Miner, abbreviated as *KNIME* [9], is an open-source data analysis platform implemented in Java and based on the IDE *Eclipse* [13]. It allows programmers to extend its basic functionality by adding so-called nodes. In order to create a new node, at least three interfaces must be implemented in Java: (i) a model class that contains the data structure of the node and provides its functionality, (ii) view classes that visualize the results once the node was executed and (iii) a dialog class used to visualize the parameters of the node and to allow the user to change them.

One disadvantage for node developers is that the design of the dialog is labor-intensive, in particular for nodes that accept a lot of parameters. Another shortcoming of *KNIME* is that only Java code can be executed using the built-in functionality. Hence, wrapper classes have to be implemented in Java if a node requires external binaries or scripts. Furthermore, *KNIME* does not support distributed execution in its free version. However, two extensions can be bought that allow either workflow execution on the SGE or on a dedicated server.

Hence, the free version of *KNIME* is not suitable for the analysis of large high-throughput data. However, *KNIME* can be used by people without programming skills for the analysis of smaller datasets using predefined nodes, especially, if a GUI is required that can be used to interactively inspect and visualize the results of the analysis.

#### Snakemake

A workflow processed by *Snakemake* [10] is defined as a set of rules. These rules must be specified in *Snakemake*’s own language in a text file named *Snakefile*. Similar to *GNU Make*, which was developed to resolve complex dependencies between source files, each rule describes how output files can be generated from input files using shell commands, external scripts or native python code. At the beginning of workflow execution, *Snakemake* automatically infers the rule execution order and dependencies based on the names of the input and output files for each rule. From version 2.4.8 on, dependencies can also be declared by explicitly referring to the output of rules defined further above. Workflows can be applied automatically to a variable number of samples using wildcards, i.e. filename patterns on present files.

In *Snakemake*, there is no clear separation between the tool library and workflow definition as the command used to generate output files is defined in the rule definition itself. Starting with version 3.5.5, *Snakemake* introduced re-usable wrapper scripts e.g. around command-line tools. In addition, it provides the possibility to include either individual rules or complete workflows as sub-workflows. Thus, *Snakemake* now allows both encapsulation of integrated tools as well as quickly adding commands directly into the workflow.

By default, no new jobs are scheduled in *Snakemake* as soon as one error is detected based on the exit code of the executed command. Accordingly, the processing of the complete workflow is halted until the user fixes the problem. This is of particular disadvantage if time-consuming tasks are applied on many replicates in parallel and one error for one replicate prevents execution of tasks for other replicates. While this default mode can be overridden by the –keep-going flag, this flag has to be set when starting execution of the workflow and applies globally independent of which particular parts of the workflow caused the error. In addition, the option –restart-times allows automatically restarting jobs after failure for a predefined number of times and each rule can specify how resource constraints are adapted in case of restarts. However, this option is only useful in case of random failure or failure due to insufficient resources. If errors result from incorrect program calls or inappropriate parameter values, restarting the task will only result in the same error again. Finally, *Snakemake* is the only one of the compared WMSs that does not provide return variables that can be used as parameters in later steps.

In summary, *Snakemake* is a much improved version of *GNU Make*. Programmers will be able to create and execute own workflows using *Snakemake* once they learned the syntax and semantic of the *Snakemake* workflow definition language. However, as *Snakemake* does not offer a GUI or editor for workflow design, most experimentalists without programming skills will not be able to create their own workflows.

#### Nextflow

The idea behind the WMS *Nextflow* [25] is to use pipes to transfer information from one task to subsequent tasks. In Unix, pipes act as shared data streams between two processes whereby one process writes data to a stream and another reads that data in the same order as it was written. In *Nextflow*, different tasks communicate through channels, which are equivalent to pipes, by using them as input and output. A workflow consists of several tasks, which are denoted as processes and are defined using *Nextflow*’s own language. The commands that are executed by processes can be either bash commands or defined in *Nextflow*’s own scripting language. *Nextflow* also provides the possibility to apply a task on a set of input files that follow a specific filename pattern using a channel that is filled with the filenames at runtime.

By default, all running processes are killed by *Nextflow* if a single process causes an error. This is particularly inconvenient if tasks with long runtimes are processed (e.g. transcriptome assembly based on RNA-seq reads). However, alternative error strategies can be defined for each task before workflow execution, which allow to either wait for the completion of scheduled tasks, ignore execution errors for this process or resubmit the process. In the latter case, computing resources can also be adjusted dynamically.

In *Nextflow*, there is no encapsulation of integrated tools at all since the commands to execute are defined in the file containing the workflow. While this is advantageous for quickly executing simple tasks, reusing tasks in the same or other workflows requires code duplication. Furthermore, *Nextflow* also does not offer a GUI for workflow design, which makes it hard for beginners to create their own workflows as they must be written in *Nextflow*’s own very comprehensive programming language.

## Conclusion

In this article, we present the WMS *Watchdog*, which was developed to support the automated and distributed analysis of large-scale experimental data, in particular next-generation sequencing data. The core features of *Watchdog* include straightforward processing of replicate data, support for and flexible combination of distributed computing or remote executors and customizable error detection that allows automated identification of technical and content-related failure as well as manual user intervention.

Due to the wide use of XML, most potential users of *Watchdog* will already be familiar with the syntax used in *Watchdog* and only need to learn the semantic. This is in contrast to other WMSs that use their own syntax. Furthermore, *Watchdog*’s powerful GUI also allows non-programmers to construct workflows using predefined modules. Moreover, module developers are completely free in which software or programming language they use in their modules. Here, the modular design of the tool library provides an easy way for sharing modules by simply sharing the module folder.

In summary, *Watchdog* combines advantages of existing WMSs and provides a number of novel useful features for more flexible and convenient execution and control of workflows. Thus, we believe that it will benefit both experienced bioinformaticians as well experimentalists with no or limited programming skills for the analysis of large-scale experimental data.

## Availability and requirements

- Project name: *Watchdog*
- Homepage: www.bio.ifi.lmu.de/watchdog; Bioconda package: anaconda.org/bioconda/watchdog-wms; Docker image: hub.docker.com/r/klugem/watchdog- wms/
- Operating system: Platform independent
- Programming language: Java, XML, XSD
- Other requirements: Java 1.8 or higher, JavaFX for the GUI
- License: GNU General Public License (GPL)
- Any restrictions to use by non-academics: none

## Additional files

Additional file 1 Overview on the *Watchdog* GUI. Contains an overview on the *Watchdog* GUI for designing workflows and a step-by-step instruction on how to use it for creating a simple workflow. (PDF 1177 kb)

Additional file 2 Replicate data analysis in *Watchdog*. Describes how to use process blocks for the automated analysis of data sets with many different replicates or conditions. (PDF 126 kb)

Additional file 3 Extending *Watchdog.* Describes how to use the plugin system to extend *Watchdog* by new executors or process blocks without changing the original *Watchdog* code. (PDF 118 kb)

Additional file 4 Computational overhead of *Watchdog*. Contains an analysis of the computational overhead of *Watchdog* and *Snakemake* for executing a workflow with a variable number of samples. (PDF 157 kb)
