# Supplementary material for: Watchdog – a workflow management system for the distributed analysis of large-scale experimental data
Source: BMC Bioinformatics. 2018 Mar 13;19:97. doi: 10.1186/s12859-018-2107-4 (PMC5850912; doi:10.1186/s12859-018-2107-4)
Supplement: Supplementary file 1 — Overview on the Watchdog GUI. Contains an overview on the Watchdog GUI for designing workflows and a step-by-step instruction on how to use it for creating a simple workflow. (PDF 1177 kb) [file 12859_2018_2107_MOESM1_ESM.pdf]

Watchdog – A Workflow Management System for the Distributed Analysis of Large-scale Experimental Data

Michael Kluge and Caroline C. Friedel

Additional File 1: Overview on the Watchdog GUI

Watchdog provides a graphical user interface (GUI), the *workflow designer*, which allows creating workflows using predefined modules and easily executing them afterwards without having to learn the *Watchdog* XML schema. Workflows created in the GUI can also be executed with the command-line version of *Watchdog* and existing *Watchdog* workflows can be loaded into the GUI.

In the following, we provide an introduction on the GUI and how to use it to create and run the simple workflow described in the main manuscript. Red highlighted boxes provide you with step-by-step instructions on how to re-create the simple workflow. The simple workflow is also included with the watchdog distribution and can be found in the *examples* sub-directory of the watchdog installation directory after running *./helper_scripts/configureExamples.sh* on Linux/macOS or manually configuring the templates in *examples/templates*. Should anything be unclear, just open the XML-file (*workflow1_basic_information_extraction.xml*) in the GUI and click on tasks, properties or dependencies to see the correct configuration.

1 Initial configuration

When the *workflow designer* is started for the first time (using *./workflowDesigner.sh* on Unix systems or *java -jar jars/WatchdogDesigner.jar* on any system), the preferences window pops up (Fig. 1, also later accessible via the menu *Edit→Preferences*). As for all configuration windows, values that have to be entered are highlighted in red, all others are set to default values and marked in green. Here, only the path to the installation directory of Watchdog has to be set. If email notification should be used, the notification category also has to be configured. Once the required fields have been filled and the changes have been saved, the actual GUI opens. The configuration file is stored in ~/*.watchdogData/* for Linux or in ~/*Library/Application Support/.watchdogData/* for *macOS*.

TODO: Configure the installation directory of *Watchdog* to use the *workflow designer*.

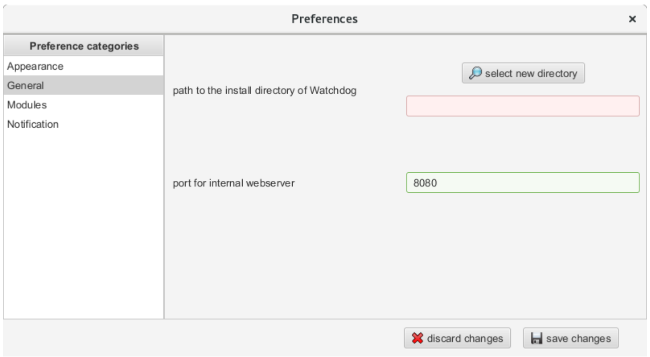

Figure 1: *Preferences window.* The left side of the window shows the categories that structure the available settings. The right side shows the settings in each category, in this case the general settings including the path to the *Watchdog* installation directory and the port used for the internal webserver (default 8080). Settings that require some changes are marked in red while valid settings are marked in green.

2 GUI overview

Fig. 2 shows the GUI after it has started. The menu at the top (①) provides core functionalities like loading, saving, validating and executing workflows as well as editing preferences. ② lists all available modules, grouped by module folders, and provides a search function on modules (*Filter modules*). By default only modules in the pre-defined module directory are loaded into the module library, but more module folders can be added in the preferences.
The central part of the window (③) is reserved for the graphical representation of the workflow. A grid structures the layout of the workflow, with each grid cell containing at most one task. A task can be added to the workflow by dragging the corresponding module from the module library, moving it to an empty grid cell and dropping it there. By clicking on the task itself, task parameters, in- or output streams and task actions can be configured in a pop-up window. At the top of each task, four rectangles visualize properties that are assigned to that task. The traffic light in the center of a task indicates the status of the task. Here, red indicates wrongly configured tasks, yellow correctly configured but unsaved tasks and green saved tasks. Workflows can be saved using *File→Save* and reloaded using *File→Open*. Saving and loading of workflows that have not been correctly configured will result in a warning.
Each task has an area for incoming (orange arrow at left side) and outgoing (blue arrow at right side) dependencies. Dependencies between tasks are created by dragging a dependency from an outgoing area to an incoming area and dropping it there. Different colors indicate task (gray) and subtask (red) dependencies and dependencies can be configured by clicking on the dashed line that represents the dependency.
④ holds property managers for creating, editing and deleting properties that can be assigned to tasks via drag and drop. Currently, four property managers are implemented that manage environment variables, executors, process blocks and constants. In order to provide a better overview of the assigned task properties and their meaning, individual colors and names can be assigned to each property. ⑤ displays status messages.
Fig. 3 illustrates the workflow design view after an example workflow has been loaded, in this case the second example workflow described in the main manuscript. The corresponding XML-file (*workflow2_differential_gene_expression.xml*) can also be found in the *examples* sub-directory of the watchdog installation directory after running *./helper_scripts/configureExamples.sh* on Linux/macOS or manually configuring the templates in *examples/templates*.

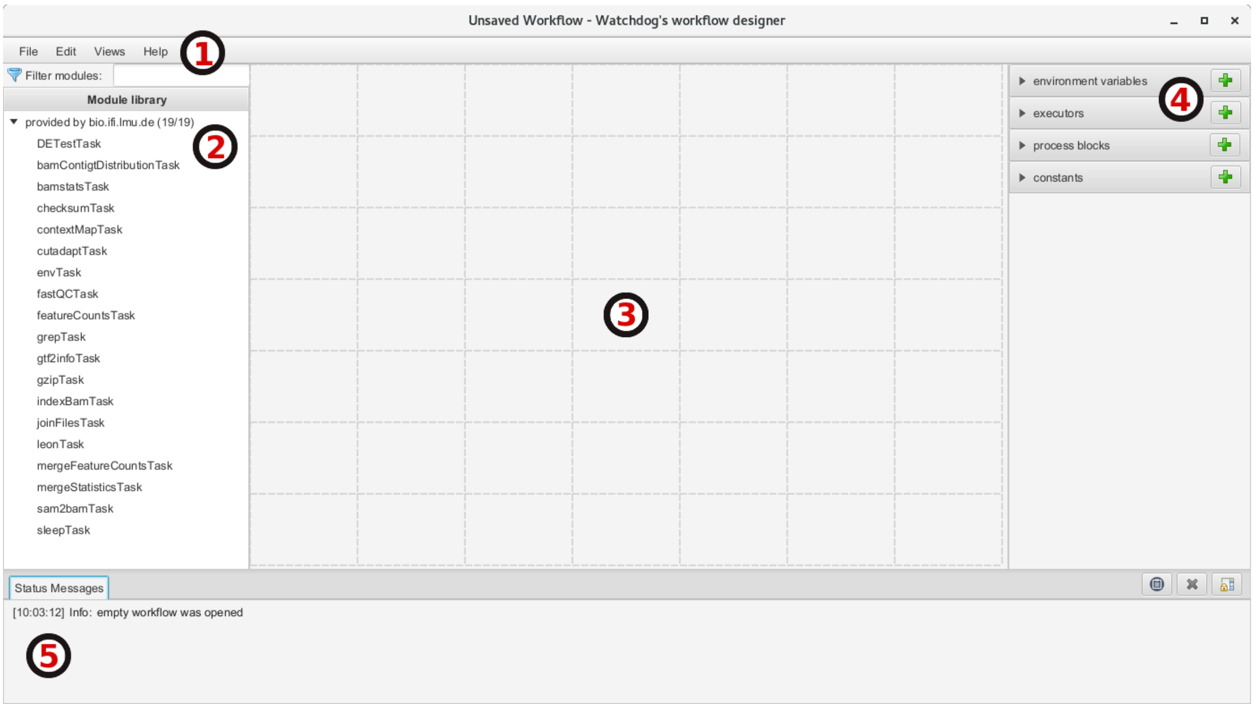

Figure 2: Layout of the *Watchdog* GUI. Numbered features are explained in the text above.

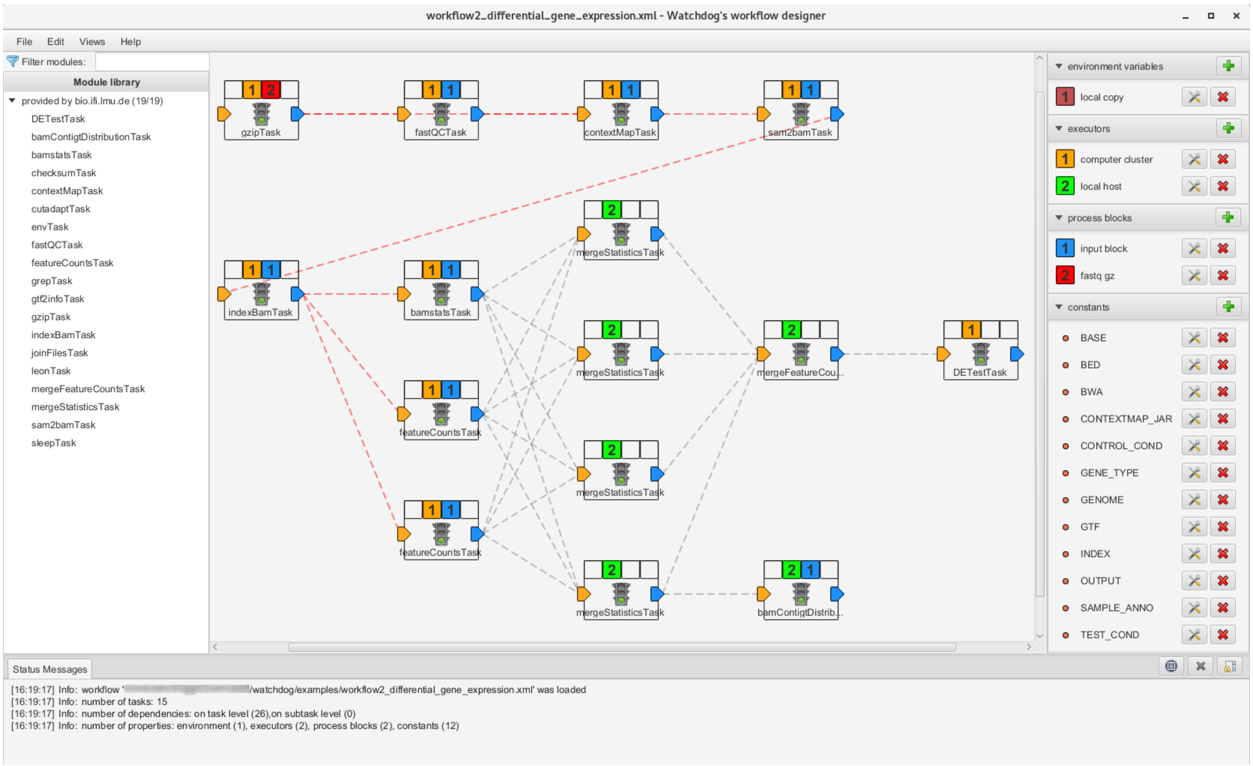

Figure 3: Workflow design view with an example workflow loaded (*workflow2_differential_gene_expression.xml*, contained in the *examples* sub-directory of the watchdog installation directory after running *./helper_scripts/configureExamples.sh*). The corresponding workflow is also described in the *Results and Discussion* section of the main manuscript. Red dashed lines indicate subtask dependencies between tasks, gray lines indicate dependencies at task level. In this case, the following tasks will be applied one after the other for each gzipped fastq files in a directory specified in the *fastq gz process block*: 1) decompress .gz files (gzipTask), 2) fastQC (fastQCTask), 3) RNA-seq mapping with ContextMap (contextMapTask), 4) conversion of resulting SAM files to BAM files using samtools sort (sam2bamTask), 5) indexing of BAM files using samtools index (indexBamTask), 6) create statistics on BAM files using samtools flagstat and samtools idxstats (bamstatsTask), 7) count reads per gene using featureCounts with different parameters (two featureCountsTasks). Subsequently, statistics files and featureCounts results for different replicates are merged (four mergeStatisticsTasks, one mergeFeatureCountsTask), differential gene expression analysis is performed (DETestTask) and distribution of reads across chromosomes and mapping statistics are visualized (bamContigDistributionTask). The workflow environment is a copy of the local environment of the user, is executed with two executors (a computer cluster and the local host), uses two process blocks (a *process input* and a *process folder*) and defines a number of constants, e.g. the base directory BASE for all analyses.

3 Defining a simple workflow

In the following sections, the functions of the GUI are explained on the example of the *basic information extraction* workflow presented in the main manuscript. Briefly, gzipped FASTQ files are decompressed, headers and sequences are extracted and stored in separate files, which are then compressed and joined.

3.1 Adding tasks

A task can be added to the workflow by dragging the corresponding module from the module library on the left side of the screen to an empty grid cell. Once the mouse enters an empty grid cell, the cell is shaded in green and the module can be dropped there (shown in Fig. 4). To find modules, the module filter above the module library can be used (Fig. 5). For this purpose, a substring search is applied on the module names. In Fig. 5, the *joinFiles* module is located using the filter function and added as last task to the workflow.
Tasks that have already been placed can be moved to other empty grid cells using drag and drop. By clicking on the task itself, task parameters, in- or output streams and task actions can be configured in a pop-up window (see Fig. 6). A right click on the task opens a context menu that allows the user to delete the task, remove in- or outgoing dependencies or delete all assigned properties.
The traffic light in the center of a task indicates the status of this task. Red traffic lights indicate incorrectly configured task (e.g. because required parameter values have not been set), yellow correctly configured but unsaved tasks and green saved tasks.

TODO: Add two *gzip*, two *grep* and one *joinFiles* task to an empty workflow.

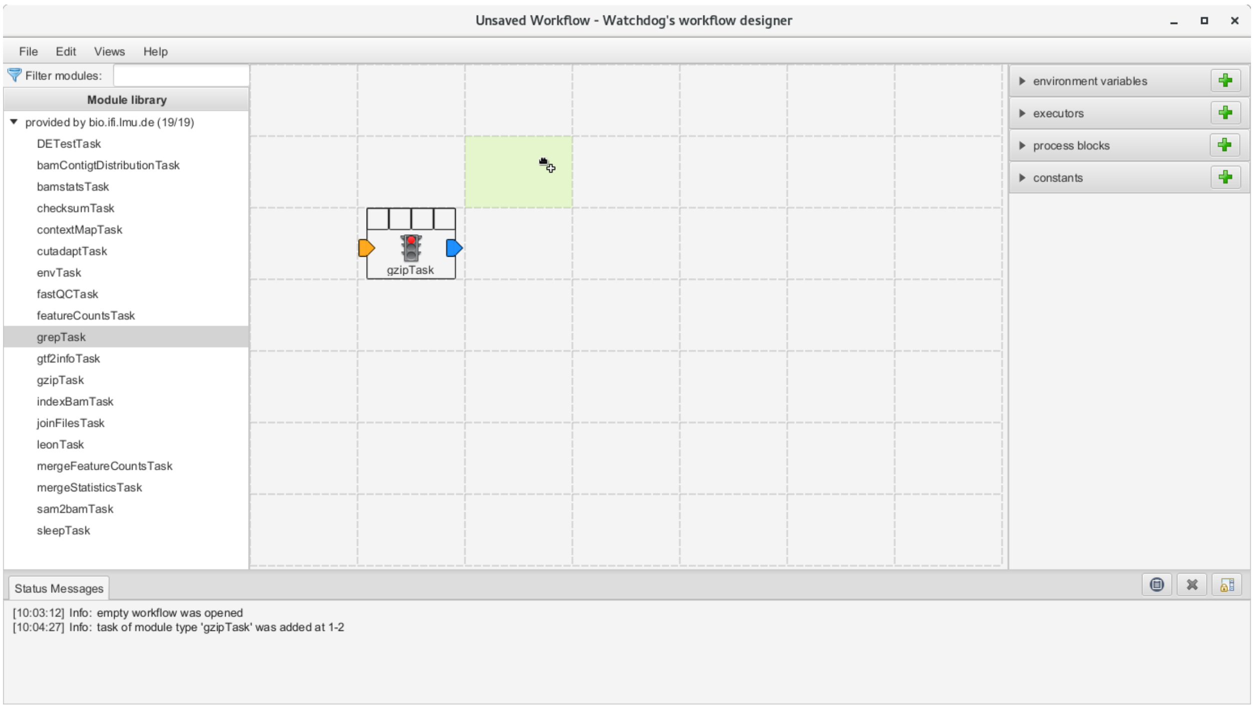

Figure 4: *Adding tasks.* The first *gzip* task has already been added to the workflow while one of the *grep* tasks is currently being dragged from the module library to its final position.

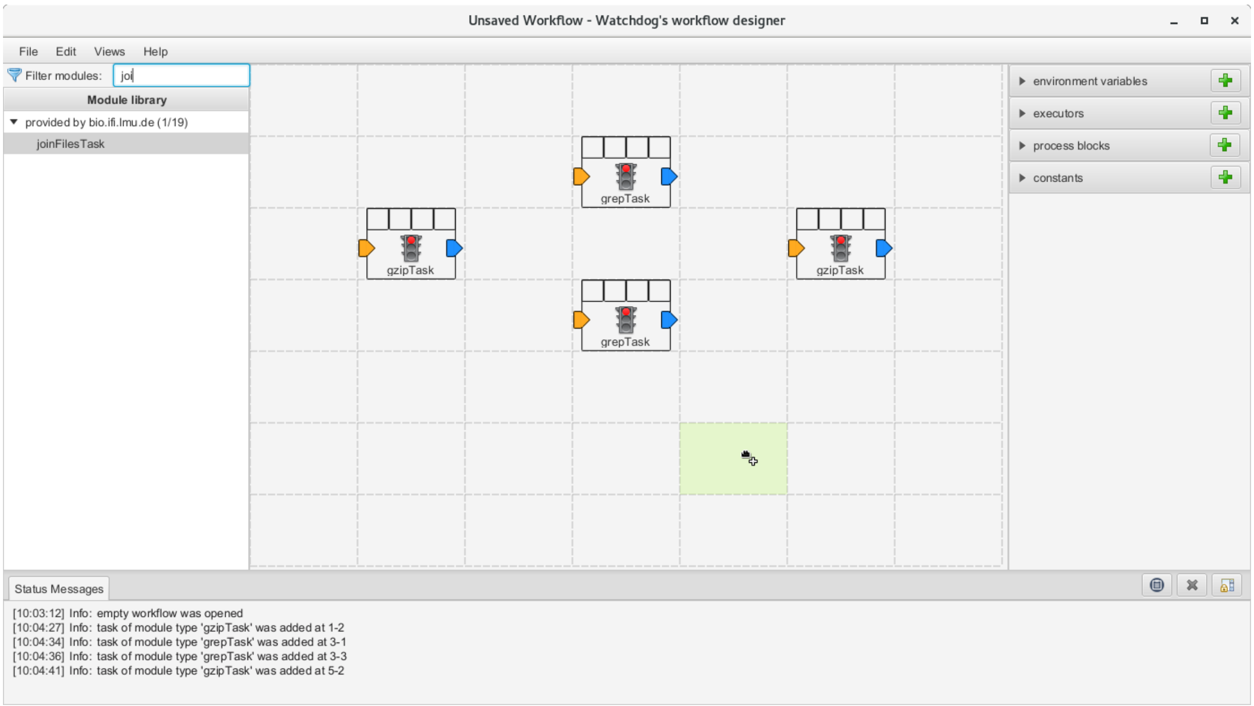

Figure 5: *Module filter.* Four of the five tasks have already been added to the workflow. The last one was located using the module filter and is currently being dragged to the cell where it should be located. The traffic lights for all tasks are red as none of the tasks have been configured.

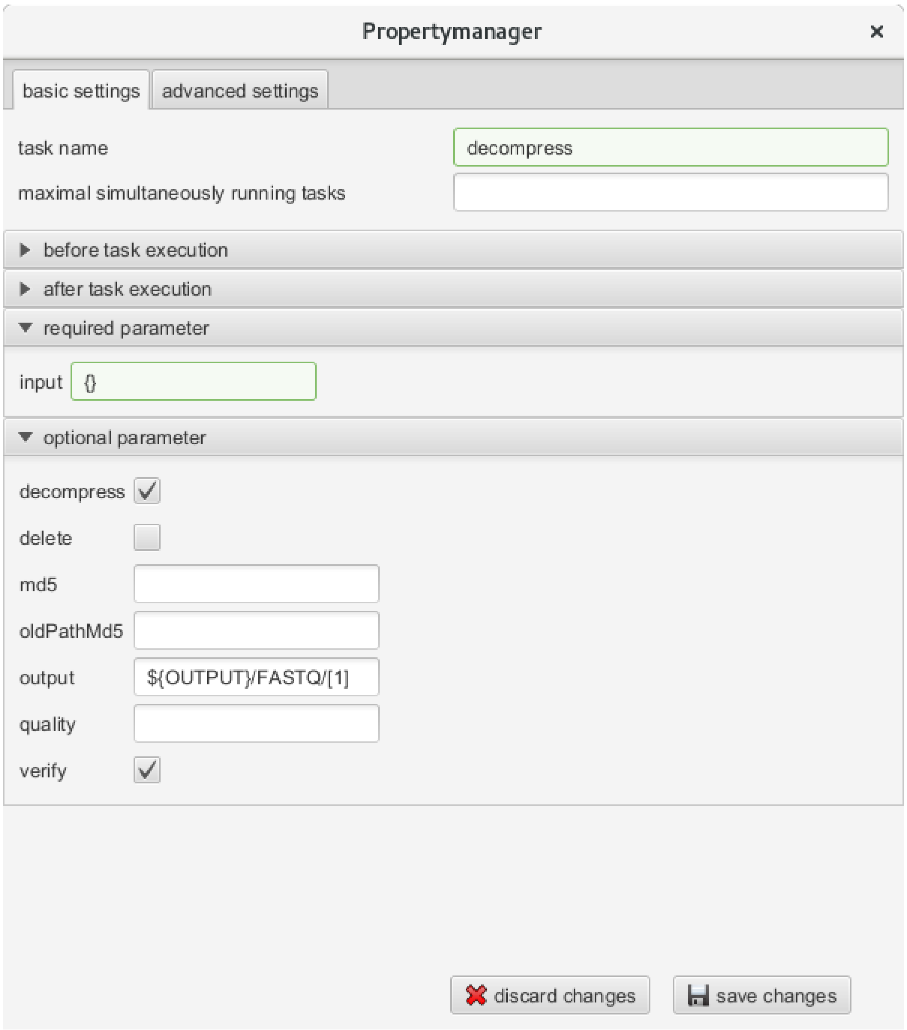

Figure 6: *Property manager for task configuration.* A left click on a task opens the configuration window for this task (in this case the first *gzip* task). Values that need to be set or changed are marked in red. Values that have been correctly set are marked in green. Here, the task name was set to *decompress*, the input is a process block variable (in this case a process folder will be used, so {} refers to an absolute file name). Furthermore, the decompress and verify options of the *gzip* task were selected. The outfile is written to the directory $\${OUTPUT}/FASTQ$ and has the same name as the original file without the *.gz* file-ending (obtained using the [1] placeholder, which returns the filename with everything after the last '.' removed). $\${OUTPUT}$ refers to a constant $OUTPUT$ defined using the *constants* property manager (see also Fig. 9 for creating constants).

3.2 Adding dependencies

Dependencies between the tasks can be added by dragging the mouse from the blue arrow of a task (e.g. (1) in Fig. 7) to the orange one of another task (e.g. (2) in Fig. 7). A dependency is visualized using a dashed line, which is gray for task dependencies and red for subtask dependencies. Dependencies can be configured by clicking on them to open the dependency properties window (see Fig. 8) and deleted using the right click menu.

> TODO: Add the dependencies shown in Fig. 7. Change the two outgoing dependencies of the first *gzip* task to subtask dependencies using the dependency properties window as shown in Fig. 8.

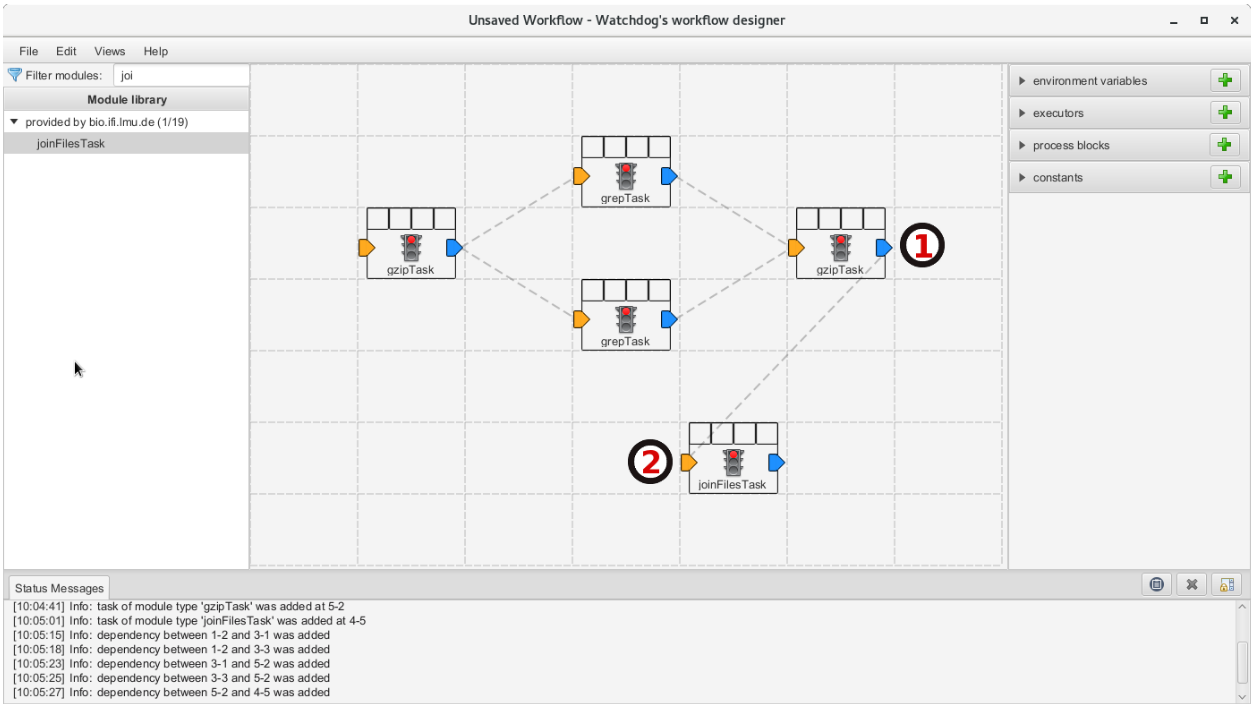

Figure 7: *Adding task dependencies.* Dependencies (dashed lines) can be added by dragging the mouse from the blue arrow of one task to the orange arrow of another task. Initially, all dependencies are at task level (gray lines). To change them to subtask dependencies (red lines) click on the dependency to open the dependency properties window (Fig. 8).

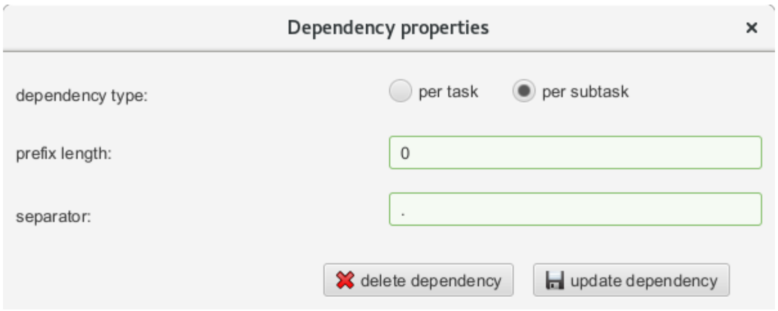

Figure 8: *Dependency properties window.* The type of the dependency (task or subtask level) can be changed using the dependency properties window that can be opened by clicking on a dependency. In case of a subtask dependency, a subtask of task B is scheduled for execution if the identifier of this subtask (defined by a process block) begins with the identifier of a finished subtask of a previous task A that task B depends on. Here, *prefix length* and *separator* determine which prefix is chosen. A prefix length of 0 indicates that the complete identifier is used. If prefix length $x > 0$, the identifier is split at the separator and the first x parts of the identifier are used as prefix (again concatenated using the separator).

3.3 Creating properties

Properties can be created by clicking on the ✚ symbol for the corresponding property. Fig. 9, Fig. 10, Fig. 11 and Fig. 12 illustrate how to create a new constant, environment, executor and process block, respectively.

TODO: Create two constants *BASE* and *OUTPUT*. *BASE* refers to the directory containing the input files (set it to *watchdog_installation_directory/examples/test_data/complex*) and *OUTPUT* to the output directory (set it to *watchdog_installation_directory/examples/workflow_output/workflow1*).

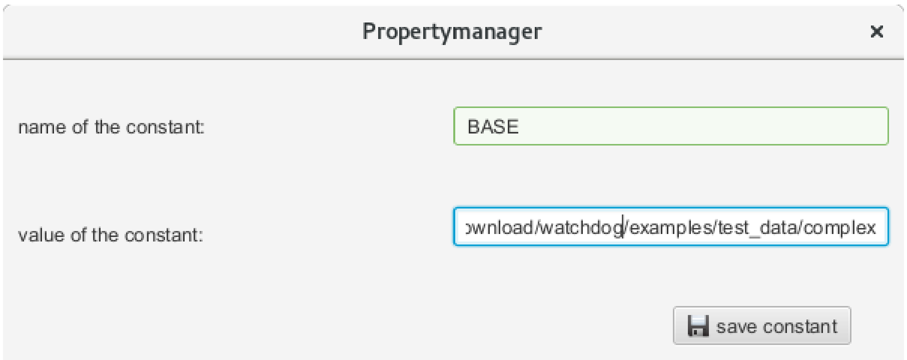

Figure 9: *Creating a new constant.* A new constant *BASE* is created and set to the value *watchdog_installation_directory/examples/test_data/complex*.

TODO: Create a new environment *local* that copies the environment of the host running Watchdog.

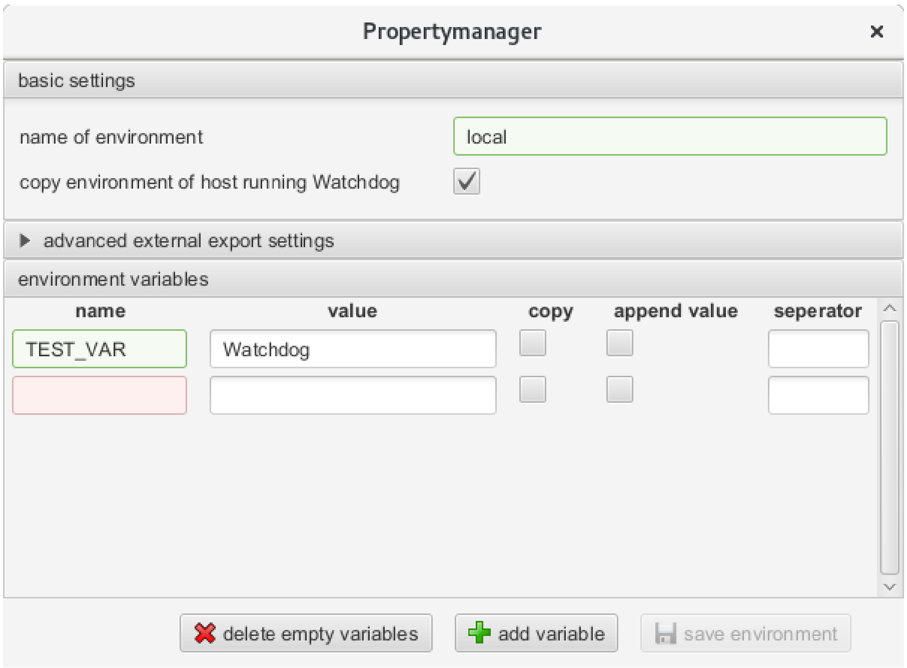

Figure 10: *Creating a new environment.* A new environment *local* is created and the environment of the host running *Watchdog* is copied to this environment. Additionally, a new environment variable *TEST_VAR* has been created and assigned the value *Watchdog*. A new empty variable has also been created which either has to be assigned a name or deleted (using *delete empty variables*) before the environment can be saved.

TODO: Create a new local executor as show in Fig. 11. As working directory select a directory of your choice. In particular, if */usr/local/storage* does not exist on your system, choose a different directory.

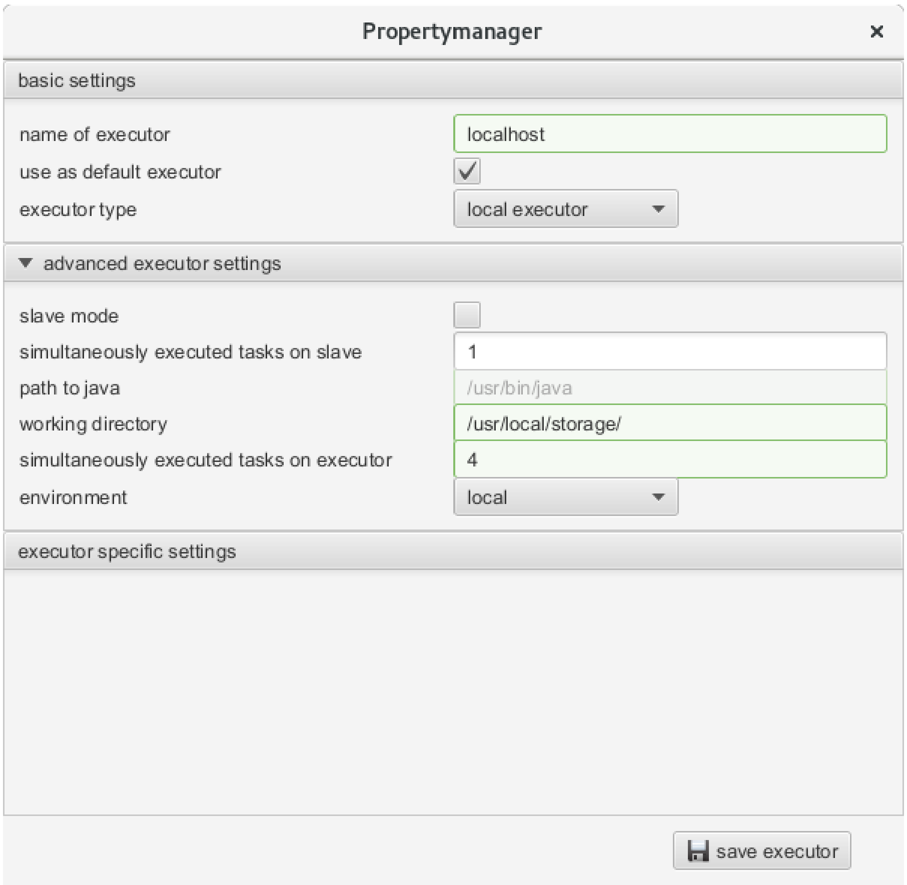

Figure 11: *Creating a new executor.* A new local executor *localhost* is created and used as default executor. The number of tasks that can be run simultaneously on this executor is set to at most 4 and the environment *local* is selected. Furthermore, the working directory is set to */usr/local/storage*. Before execution of each task, *Watchdog* changes to the working directory.

TODO: Create two process folders with name *fastq_gz* and *split* as shown in Fig. 12 and one process input with name *input*. The process folder *fastq_gz* should select all files in the directory $\$\{BASE\}/FASTQ/gz/$ matching the file pattern **fastq.gz* and process folder *split* should select all files in the directory $\$\{OUTPUT\}/split/$ matching the file pattern **.txt*. Enforce existence at runtime only for *fastq_gz*.

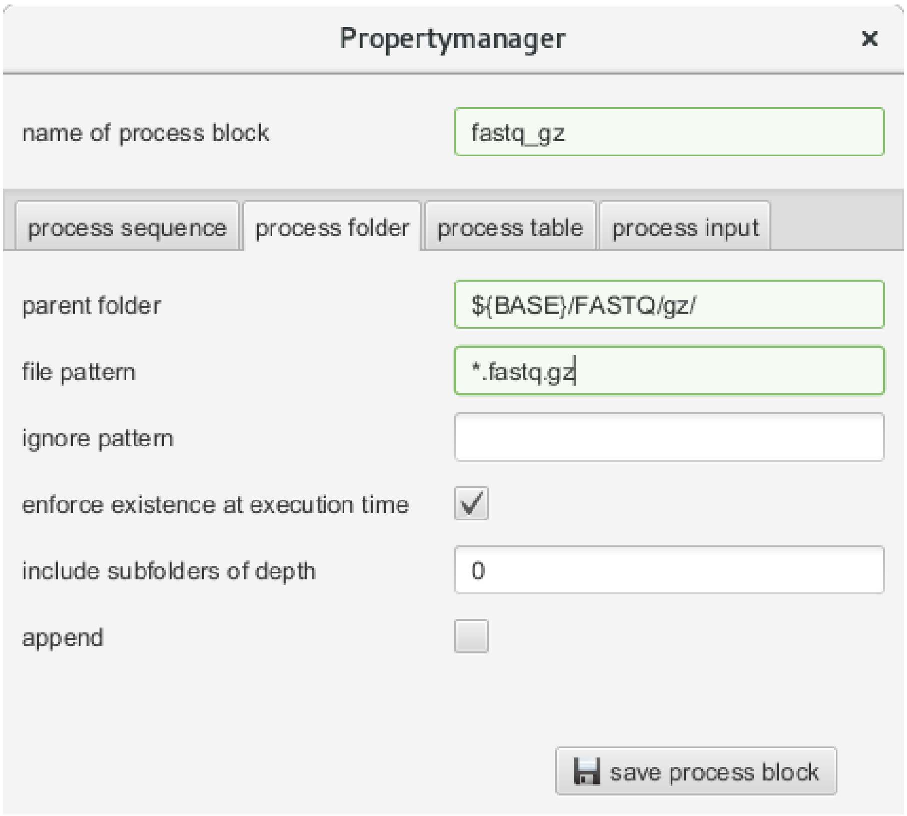

Figure 12: *Creating new process blocks.* A new process folder *fastq_gz* is created that selects all files in the directory $\$\{BASE\}/FASTQ/gz/$ matching the file pattern **.fastq.gz*. Existence of this folder is enforced at execution time, which means that the folder will be created if it does not yet exist. No sub-folders will be included (depth 0).

3.4 Assigning properties to tasks

Environments, executors or process blocks can be assigned as properties to tasks via drag and drop (see Fig. 13). The first square on top of each task holds the environment, the second the executor, the third the process block while the last square is currently not in use.

TODO: Assign all properties as shown in Fig. 14 using drag and drop.

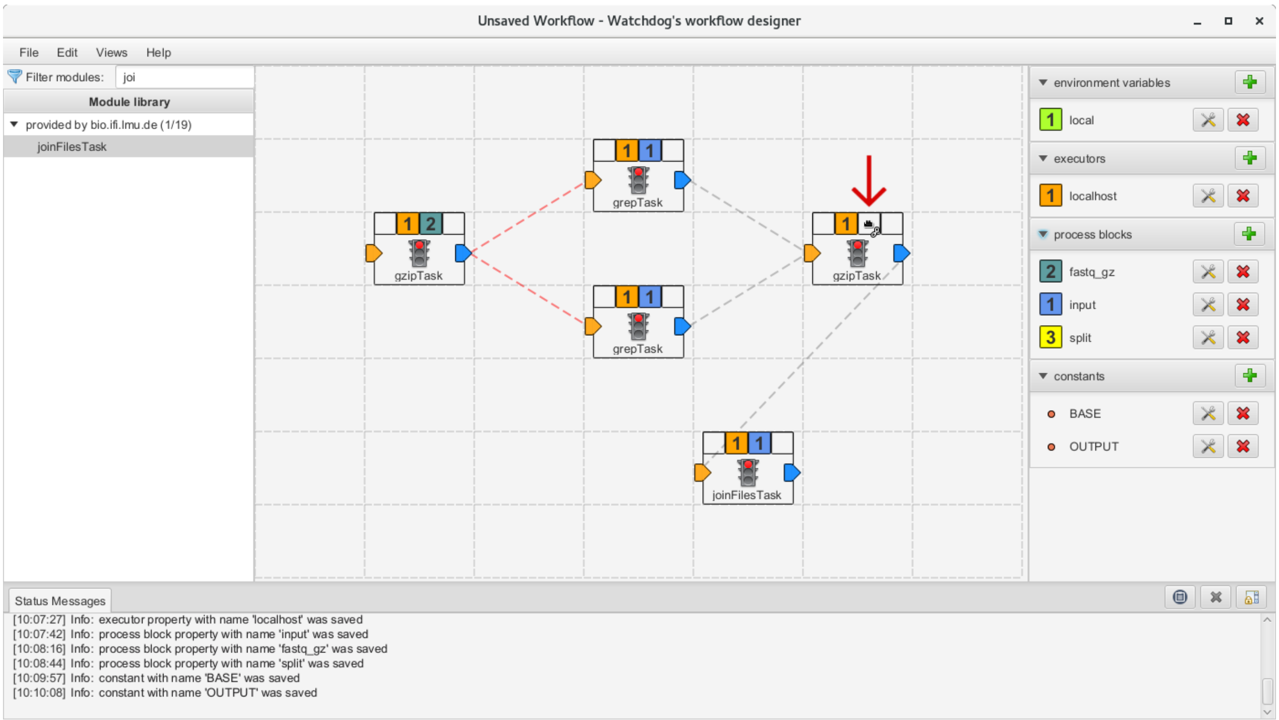

Figure 13: *Assigning properties to tasks.* All required properties have already been assigned except the *split* process block (yellow) for the second *gzip* task, which is currently being dragged over its designated square (indicated by a red arrow).

4 Validate & Execute

If all tasks have been configured correctly (for task configuration see Fig. 6), the traffic light of all tasks turns orange (see Fig. 14). You can now validate (*Edit*→*Validate workflow*) and save (*File*→*Save*) the workflow. Saving will automatically validate the workflow and turn all task traffic lights to green (see Fig. 15). Incorrectly configured workflows can also be saved and reloaded, but this will trigger warnings in both cases as they cannot be validated. Thus, it is recommended to save any intermediate workflow that has been configured correctly.

To execute a validated workflow, switch to the execution view (*Views*→*Execution view*) and click execute at the bottom of the GUI (see Fig. 16). While the workflow is executed, the hourglass symbol indicates running or waiting tasks, ✔ indicates a successfully finished task and ✕ a failed task. Workflow execution can be stopped or scheduling be paused via the GUI. Manual intervention into execution of individual tasks is possible via the web-interface. The link to the web-interface will either be shown in the execution log window or sent by email if email notification is enabled.

TODO: Configure all tasks. The values assigned to parameters for each task can be found in the XML-file *workflow1_basic_information_extraction.xml*. Some of these are optional and can only be edited after clicking on the arrowhead beside *optional parameter*. Validate, save and execute the workflow.

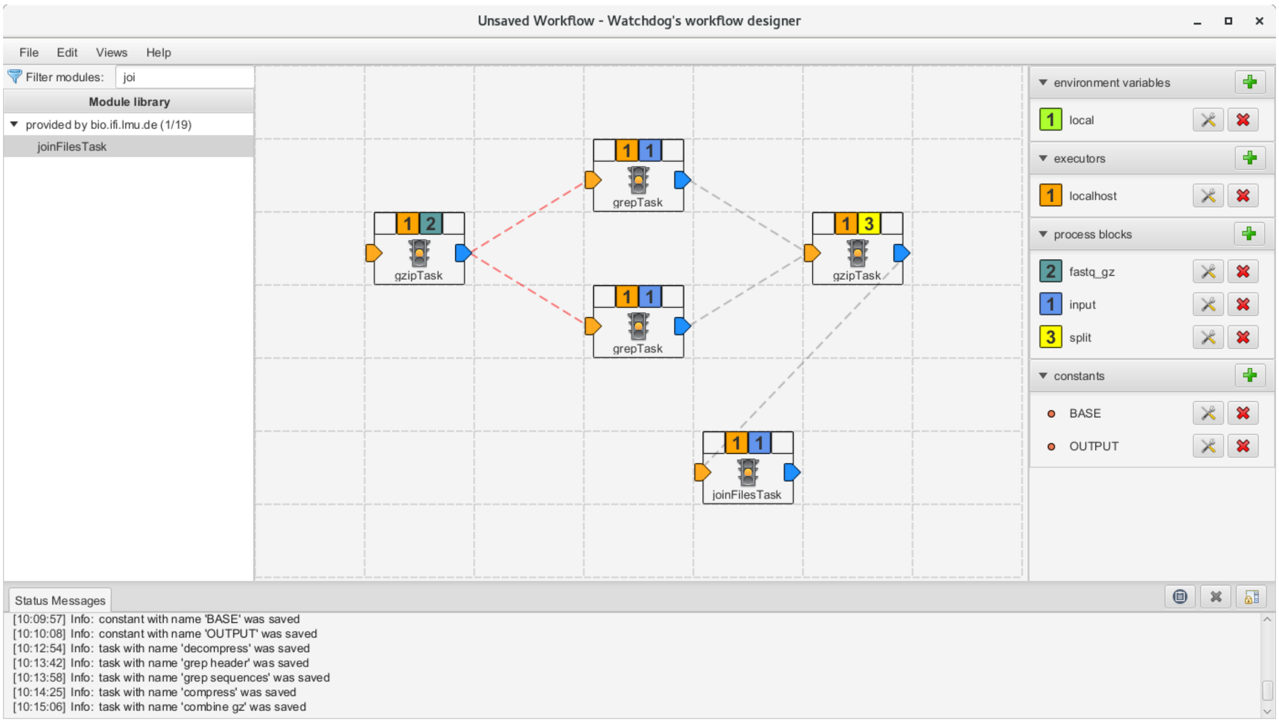

Figure 14: If all tasks are configured correctly, their traffic lights are orange and the workflow can be validated.

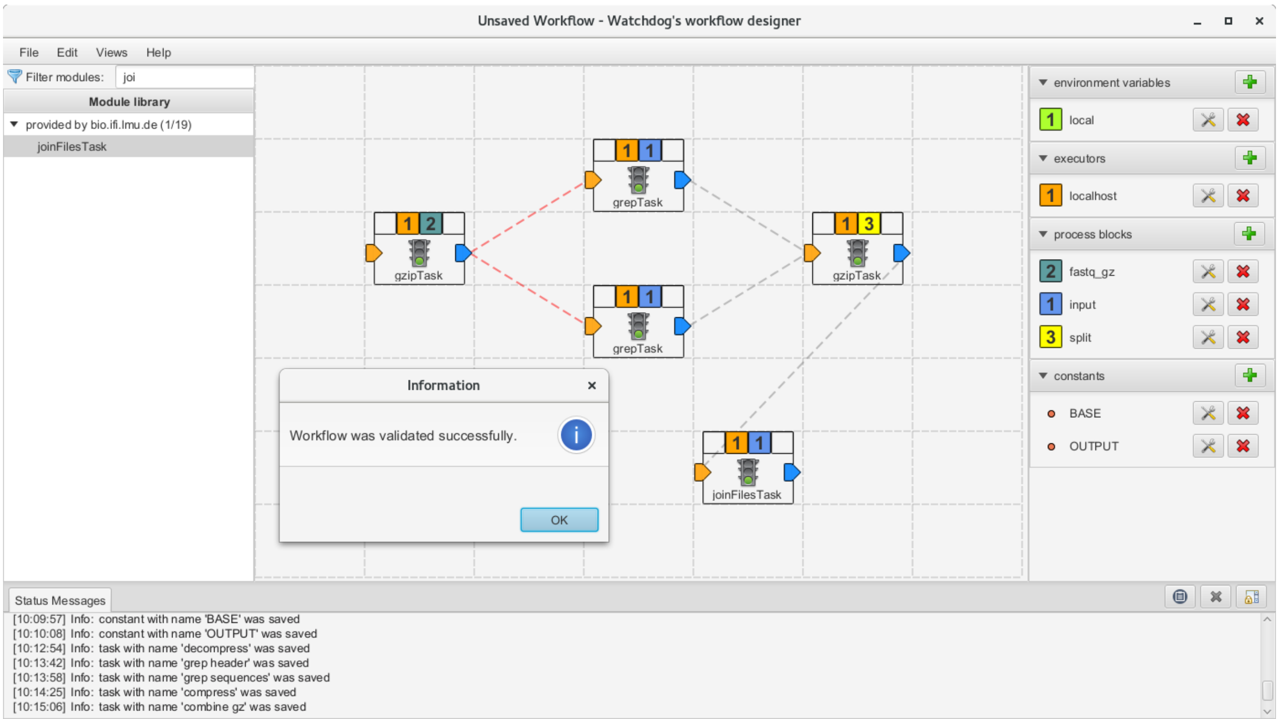

Figure 15: If the workflow has been validated successfully, all traffic lights turn to green and it can be saved without warnings. Here, the workflow was already saved before running validate again.

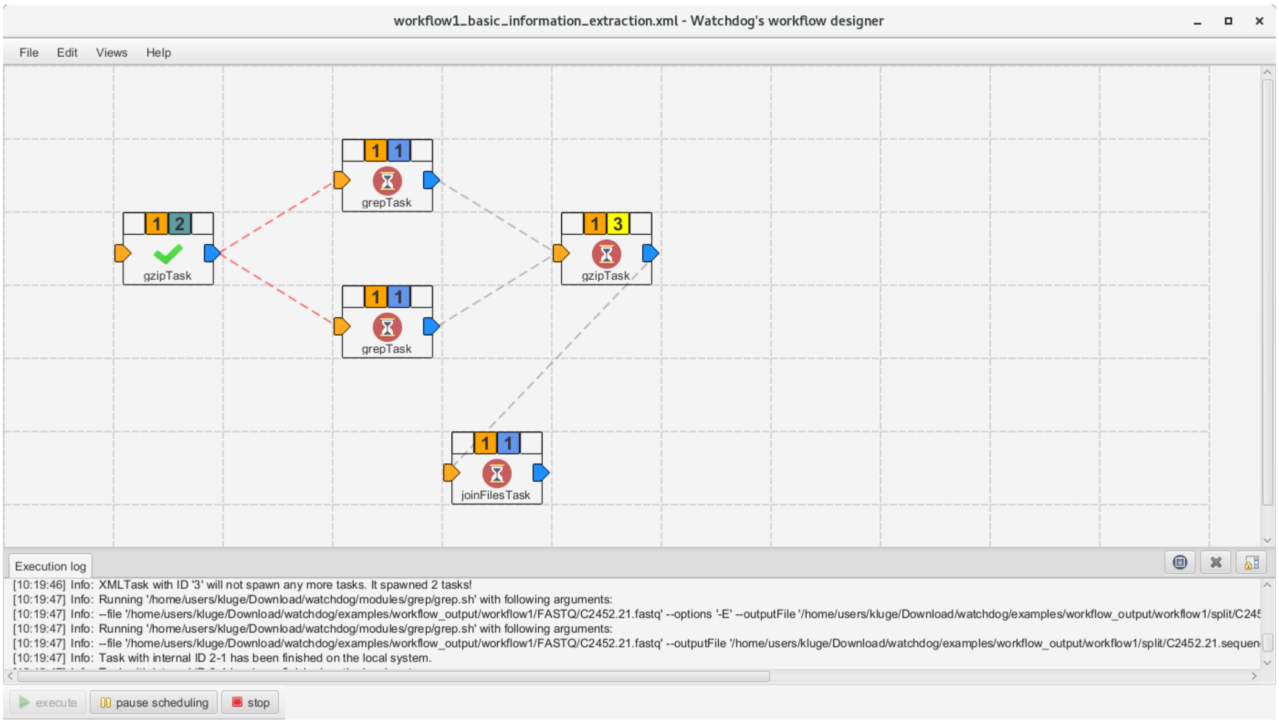

Figure 16: The execution of the workflow has been started and the first *gzipTask* has already finished successfully. All other tasks are either running or waiting.

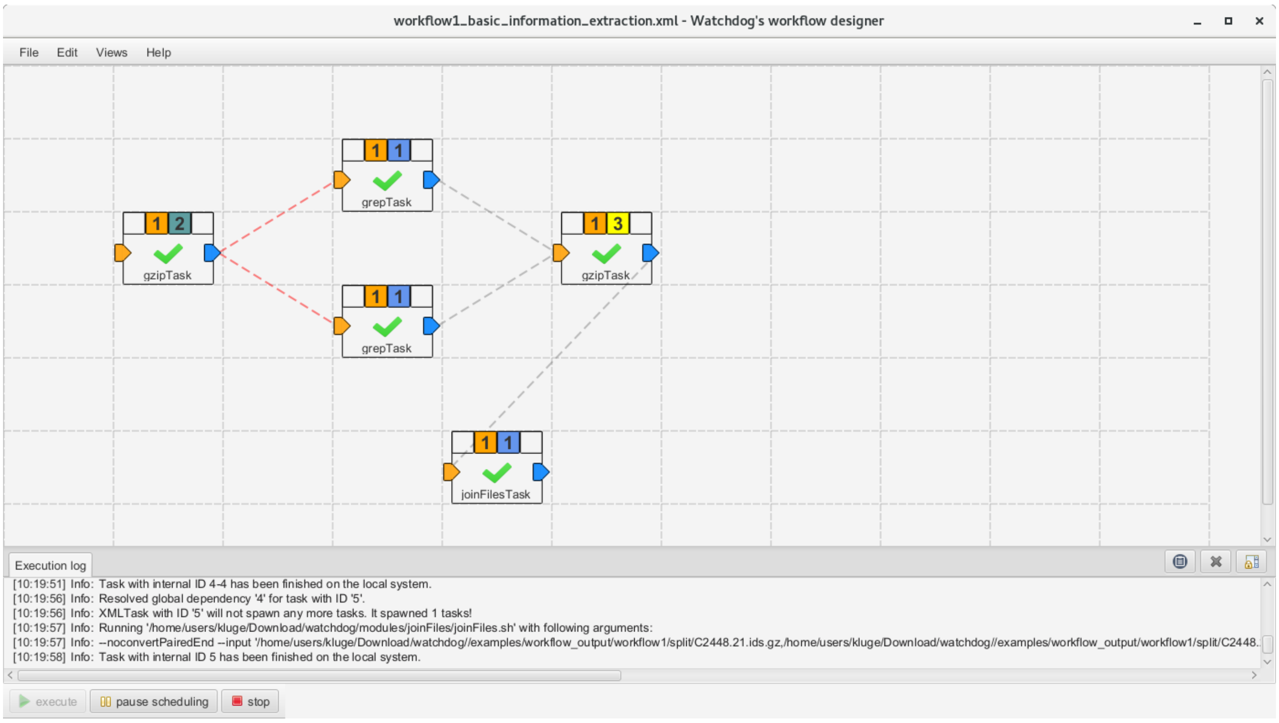

Figure 17: The execution of the workflow finished successfully.
