# Supplementary material for: Watchdog – a workflow management system for the distributed analysis of large-scale experimental data
Source: BMC Bioinformatics. 2018 Mar 13;19:97. doi: 10.1186/s12859-018-2107-4 (PMC5850912; doi:10.1186/s12859-018-2107-4)
Supplement: Supplementary file 2 — Replicate data analysis in Watchdog. Describes how to use process blocks for the automated analysis of data sets with many different replicates or conditions. (PDF 126 kb) [file 12859_2018_2107_MOESM2_ESM.pdf]

Watchdog – A Workflow Management System for the Distributed Analysis of Large-scale Experimental Data

Michael Kluge and Caroline C. Friedel

Additional File 2: Replicate data analysis in Watchdog

1 Introduction

In the following, a short introduction is provided on how to use process blocks in *Watchdog* to analyze data sets with multiple samples. For this purpose, we will use an example experiment in which sequencing has been performed for 9 conditions with 2 replicates each. We assume that the corresponding gzipped FASTQ files are contained in a directory named */usr/local/storage/examples/FASTQ/zipped/* and the file names for the corresponding 18 files are:

```
C2439_ATCACG_L001.fastq.gz
C2440_GAGTGG_L001.fastq.gz
C2441_AGTCAA_L001.fastq.gz
C2442_ACAGTG_L001.fastq.gz
C2443_GTGAAA_L001.fastq.gz
C2444_ATGTCA_L001.fastq.gz
C2445_CGATGT_L002.fastq.gz
C2446_GATCAG_L002.fastq.gz
C2447_TTAGGC_L002.fastq.gz
C2456_ATTCCT_L003.fastq.gz
C2457_ACTGAT_L004.fastq.gz
C2458_TGACCA_L004.fastq.gz
C2459_CAGATC_L004.fastq.gz
C2460_GTTTCG_L004.fastq.gz
C2461_CCGTCC_L004.fastq.gz
C2462_GGCTAC_L004.fastq.gz
C2463_ATCACG_L005.fastq.gz
C2464_GAGTGG_L005.fastq.gz
```

We will show how to use either a process block or process table in combination with a process input to decompress these files and run FastQC for them. For this purpose, we first need to define the corresponding process blocks within the *settings* environment of a *Watchdog* workflow:

```
1  <settings>
2      <constants>
3         <const name="BASE">/usr/local/storage/examples/</const>
4      </constants>
5      <processBlock>
6         <processFolder name="fastq_gz" folder="${BASE}/FASTQ/zipped/"
             pattern="*.fastq.gz"/>
7         <processTable name="table" table="${BASE}/sample_name_table.txt"/>
8         <processInput name="input_block"/>
9      </processBlock>
10 </settings>
```

Line 3 defines the constant BASE containing the path to the directory in which data and results are stored. Line 6 defines a process folder containing all files with the ending *.fastq.gz* within the *FASTQ/zipped/* subdirectory of the BASE directory. Line 7 defines a process table described by the tab-separated file *sample_name_table.txt* (for details see section 5). Finally, line 8 defines a process input block.

2 Accessing process block variables

To create subtasks based on process blocks, special placeholders used in the task definitions are replaced with the values of variables stored in instances of the process block. For example, the value of the variable with the name *type* of the process table in Fig. 4c in the main manuscript is accessed with $\{\$type\}$. In case of process blocks with only a single variable, the name of the variable is omitted (see Fig. 6 in the main manuscript). Independent of the type of the process block, the user has the possibility to obtain a prefix of a variable value using an arbitrary character as separator. For example $\{\$file, 1, .\}$ removes everything after the last period (i.e. the file-ending) of a variable with the name $\$file$. If no separator is indicated, '.' is used by default. If the value stored in a variable is a file path, two additional selectors are available: [] returns the name of the file whereas () delivers the absolute path to the parent folder of the file. For more details, please refer to the manual.

3 Using a process folder

If we now want to decompress all *fastq.gz* files for the experiment, we can define the following task in the *tasks* environment of the workflow:

```
<gzipTask id="1" name="extract" processBlock="fastq_gz">
    <parameter>
       <decompress>true</decompress>
       <delete>false</delete>
       <input>{}</input>
       <output>${BASE}/FASTQ/unzipped/[1]</output>
    </parameter>
</gzipTask>
```

This creates one subtask for each *fastq.gz* file in the directory and the resulting decompressed files are written to the *FASTQ/unzipped* subdirectory of the BASE directory. Here, the absolute file path of the file is accessed with {}, while [1] returns only the filename with everything after the last '.' removed. Thus, for the first file in the example {} returns */usr/local/storage/examples/FASTQ/zipped/C2439_ATCACG_L001.fastq.gz*, while [1] returns *C2439_ATCACG_L001.fastq*.

The selection of files can be modified easily by modifying the pattern in the definition of the process folder. For instance, the following would select only the first nine samples:

```
        <processFolder name="fastq_gz" folder="${BASE}/FASTQ/zipped/"
           pattern="C24(39|4[0-7])*.fastq.gz"/>
```

If some *fastq.gz* files are contained in sub-directories of *FASTQ/zipped/*, they can also be selected by using the *maxDepth* attribute of the process folder block. For instance, the following would select all files in the *FASTQ/zipped/* subdirectory as well as its direct sub-directories:

```
        <processFolder name="fastq_gz" folder="${BASE}/FASTQ/zipped/"
           pattern="*.fastq.gz" maxDepth="1"/>
```

4 Using a process input

While we could reuse the process folder defined above or create a new process folder for the decompressed files in order to perform FastQC for those files, process input blocks provide a simpler way. Using process input blocks, one subtask of task B will be created for every successfully finished subtask of a previous task A that B depends on. Thus, they can only be used for tasks that depend on previous tasks. For our example, the *fastQCTask* could be created using the process input block in the following way:

```
<fastQCTask id="2" name="fastqc" processBlock="input_block">
    <dependencies>
       <depends separate="true">1</depends>
    </dependencies>
    <parameter>
```

```
        <fastq>{$createdGzipFile}</fastq>
        <outdir>${BASE}/fastqc/</outdir>
    </parameter>
</fastQCTask>
```

For each decompressed *fastq* file, FastQC is called and the results are written to the *fastqc* subdirectory of the BASE directory. Return values of the previous task can be accessed as {*$return_value*}. In this case, the absolute file path of the decompressed *fastq* file is stored in the return value *createdGzipFile* of the *gzipTask* and accessed by the *fastQCTask* as {*$createdGzipFile*}. Furthermore, subtask dependencies are used (using the *separate* attribute of the *dependencies* element). Thus, as soon as decompression of one *fastq.gz* file has finished, the corresponding FastQC task is scheduled even if the other *fastq.gz* files have not been decompressed yet.

5 Using a process table

The file names in the provided example are quite uninformative. Instead of renaming the raw input files or the final output files by hand or a separate script, all analysis files can be automatically named according to condition and replicate using a process table. For instance, assume we have the following tab-separated file *sample_name_table.txt* with three columns, one for the uninformative sample identifier, one for the condition and one for the replicate:

```
SAMPLE_ID COND REP
C2439_ATCACG_L001 Cond1 1
C2440_GAGTGG_L001 Cond2 1
C2441_AGTCAA_L001 Cond3 1
C2442_ACAGTG_L001 Cond4 1
C2443_GTGAAA_L001 Cond5 1
C2444_ATGTCA_L001 Cond6 1
C2445_CGATGT_L002 Cond7 1
C2446_GATCAG_L002 Cond8 1
C2447_TTAGGC_L002 Cond9 1
C2456_ATTCCT_L003 Cond1 2
C2457_ACTGAT_L004 Cond2 2
C2458_TGACCA_L004 Cond3 2
C2459_CAGATC_L004 Cond4 2
C2460_GTTTCG_L004 Cond5 2
C2461_CCGTCC_L004 Cond6 2
C2462_GGCTAC_L004 Cond7 2
C2463_ATCACG_L005 Cond8 2
C2464_GAGTGG_L005 Cond9 2
```

Using the process table defined in section 1 based on this file, we can then name the decompressed *fastq* files according to condition and replicate in a straightforward way in the task definition:

```
<gzipTask id="1" name="extract" processBlock="table">
    <parameter>
        <decompress>true</decompress>
        <delete>false</delete>
        <input>${BASE}/FASTQ/zipped/{$SAMPLE_ID}.fastq.gz</input>
        <output>${BASE}/FASTQ/unzipped/{$COND}_{$REP}.fastq</output>
    </parameter>
</gzipTask>
```

Here, one *gzipTask* is created for each row of the table. For each subtask, the values of the variables (variable names provided in the first line of the file) can be accessed as {*$variable_name*}. Thus, {*$SAMPLE_ID*} accesses the value of the first column, {*$COND*} the value of the second column and {*$REP*} the value of the third. In this example, the file *C2439_ATCACG_L001.fastq.gz* would be decompressed to file *Cond1_1.fastq*, the file *C2440_GAGTGG_L001.fastq.gz* to file *Cond2_1.fastq*, and so on.

The subsequent FastQC tasks can then either be created using the input process block as described in the previous section or by reusing the process table or creating a new process folder.
