# Supplementary material for: Watchdog – a workflow management system for the distributed analysis of large-scale experimental data
Source: BMC Bioinformatics. 2018 Mar 13;19:97. doi: 10.1186/s12859-018-2107-4 (PMC5850912; doi:10.1186/s12859-018-2107-4)
Supplement: Supplementary file 3 — Extending Watchdog. Describes how to use the plugin system to extend Watchdog by new executors or process blocks without changing the original Watchdog code. (PDF 118 kb) [file 12859_2018_2107_MOESM3_ESM.pdf]

# Watchdog – A Workflow Management System for the Distributed Analysis of Large-scale Experimental Data

Michael Kluge and Caroline C. Friedel

***Additional File 3: Extending Watchdog***

## 1 Introduction

*Watchdog* provides a flexible plugin system that allows extending *Watchdog* by additional types of executors and process blocks without modifying the original Java classes. Essentially, this means creating a new XML element for use in *Watchdog* workflows as well as implementing additional Java classes that provide the functionality for this element.

In brief, you have to do the following to use the plugin system:

- Create an XSD file describing the new element and its parent element for use in *Watchdog* workflows
- Extend a few abstract classes
- Add class files for the new classes to the *Watchdog* jar-file and copy the new XSD file to a sub-directory of the *Watchdog* installation directory

In the *Watchdog* command-line version, all non-abstract classes in the *Watchdog* jar-file that extend the *XMLParserPlugin* abstract class are loaded dynamically during workflow execution. Currently, this is restricted to XML parsers for the generic type *ProcessBlock* or *ExecutorInfo*. The XML parser for a new XML element provides the functionality to parse this element in a workflow (i.e. a new executor or process block type) and to create a new object representing the corresponding element type. Here, the four most important functions of the *XMLParserPlugin* abstract class that have to be implemented are:

- getNameOfParseableTag: returns the name of the element the class can parse
- getNameOfParentTag: returns the name of the parent element of this element
- getXSDDefinition: returns the path to the XSD file describing this element (relative to the *xsd* sub-directory of the *Watchdog* directory)
- parseElement: implements the actual parsing process.

The last function creates an object of a class representing the new element. This class has to implement the interfaces XMLDataStore and XMLPlugin, for instance by extending one of the abstract classes *ProcessBlock* or *ExecutorInfo* or one of their subclasses.

For use in the Workflow designer GUI of *Watchdog*, two additional requirements have to be met:

- An FXML file has to be provided describing how the attributes of the new element type are represented graphically. FXML is an XML-based markup language for describing the layout of a user interface in a JavaFX application.
- Classes extending *PluginView* and *PluginViewController* have to be implemented for testing whether the input is valid and for loading and saving data to and from XML.

# 2 Example: Adding a new executor type to Watchdog

In the following, we will describe for an example how to implement a new executor type for *Watchdog* using the plugin system. In this example, a new executor is implemented for executing tasks on supercomputers or computer clusters running the Slurm Workload Manager. This executor is now also provided with *Watchdog*. In this case, a lot of the functionality is already covered by abstract classes representing externally scheduled executors. These abstract classes are also used in the implementation of the cluster executor providing DRMAA support. Section 3 describes how this new Slurm executor can be used in a workflow.

## 2.1 Create the XSD definition for Slurm

In the first step, the new XML element for the executor, its parent and attributes have to be described in an XSD file (see below). Here, the element name is *slurm* (line 20) and the element is of type *executorSlurmType*, which is described in lines 5-17. The attribute *substitutionGroup* (line 20) indicates that its parent is of type *executorType*. Thus, this new element can be used as a child of the *executor* element. The *executorSlurmType* extends the type *executorAbstractType* (line 7), which means that it inherits all of its attributes. In addition, it overwrites the *type* attribute of the *executorAbstractType* (line 8) and has a number of specific attributes (lines 9-14).

```
1  <!-- part of file 'xsd/plugins/executor.slurm.xsd' -->
2  <!-- definition of custom types not shown -->
3
4  <!-- slurm executor -->
5  <x:complexType name="executorSlurmType">
6       <x:simpleContent>
7            <x:extension base="executorAbstractType">
8                 <x:attribute name="type" type="x:string" fixed="slurm"/>
9                 <x:attribute ref="cpu" default="1"/>
10                <x:attribute ref="memory" default="3000M"/>
11                <x:attribute name="cluster" type="x:string"/>
12                <x:attribute name="disableDefault" type="x:boolean"/>
13                <x:attribute name="customParameters" type="x:string"/>
14                <x:attribute ref="timelimit" default="0-12:0"/>
15           </x:extension>
16      </x:simpleContent>
17 </x:complexType>
18
19 <!-- tag for executor in XML file -->
20 <x:element name="slurm" type="executorSlurmType" substitutionGroup="
       executorType"/>
```

## 2.2 Implement the class representing the new element

In the second step, the class representing the new element (*SlurmExecutorInfo*) has to be implemented by implementing the *XMLDataStore* and *XMLPlugin* interfaces. Important parts of the corresponding code are shown below. For the rest of this section, red text within code listings highlights parts that are specific for the Slurm executor and would have to be adjusted if a different type of executor would be implemented, while black text could simply be copied.

In this example, the abstract class *ExternalExecutorInfo* is extended, which implements the necessary interfaces and provides common functionalities of external executors. The important abstract functions that have to be overwritten are:

- getExecutorForTask: creates the Executor for this class (lines 37-39), in this case a new object of class *SlurmExecutor* (implementation described in section 2.4).
- toXML: implements how to write the element in XML format (lines 19-34). This function is called for the element if the workflow is written to XML (e.g. in the GUI).

In addition, the constructor (not shown below) and getter functions (only shown for the CPU attribute, line 14-16) for the new attributes (lines 5-10) have to be implemented.

```
public class SlurmExecutorInfo extends ExternalExecutorInfo {

    ...

    private final String CUSTOM_PARAMS;
    private final boolean IGNORE_DEFAULT_PARAMS;
    private final int CPU;
    private final String MEMORY;
    private final String CLUSTERS;
    private final String TIMELIMIT;

    ...

        public int getCPUs() {
        return this.CPU;
    }

    @Override
    public String toXML() {
        XMLBuilder x = new XMLBuilder();
        // start with basic tag
        x.startTag(SlurmWorkloadManagerConnector.EXECUTOR_NAME, false);
        x.addQuotedAttribute(XMLParser.NAME, this.getName());

        // add optional attributes
        if(this.hasDefaultEnv())
            x.addQuotedAttribute(XMLParser.ENVIRONMENT, this.getEnv().getName());

        ...

        // end the tag
        x.endCurrentTag();
        return x.toString();
    }

    @Override
    public Executor<SlurmExecutorInfo> getExecutorForTask(Task t, SyncronizedLineWriter logFile) {
        return new SlurmExecutor(t, logFile, 1, this);
    }

    ...
}
```

## 2.3 Implement XML Parser for the new element

As a next step, an XML Parser class implementing the *XMLParserPlugin* has to be created. In this case, we simply extend the abstract class *XMLExecutorInfoParser*. Here, the main work has to be done for the implementation of the parseElement function, which obtains the attributes from the XML element and creates and returns a new *SlurmExecutorInfo* object. For this purpose, the function getXSDDefinition (lines 40-42) has to return the path for the XSD file for this element (relative to the *xsd* sub-directory of the *Watchdog* installation directory). Before using the new executor, the XSD file has to be copied to this place. We recommend using the *xsd/plugins* sub-directory for this purpose.

```java
public class SlurmExecutorInfoParser extends XMLExecutorInfoParser<SlurmExecutorInfo> {

    //This is were the XSD file for the new element has to be
    private static final String XSD_DEF = "plugins" + File.separator + "executor.slurm.xsd";

    static {
        // set monitor thread on Executor
        SlurmMonitorThread.updateMonitorThread();

        // register the executor plugins shipped with watchdog on GUI
        ExecutorPropertyViewController.addNewType(SlurmExecutorInfo.class, SlurmGUIExecutorView.class);
    }

    public SlurmExecutorInfoParser(Logger l) {
        super(l);
    }

    @Override
    public String getNameOfParseableTag() {
        return SlurmWorkloadManagerConnector.EXECUTOR_NAME;
    }

    @Override
    public SlurmExecutorInfo parseElement(Element el, String watchdogBaseDir, Object[] additionalData) {
        DefaultExecutorInfo di = this.parseMandatoryParameter(el, watchdogBaseDir, additionalData);

        // get additional slurm attributes
        int cpu = Integer.parseInt(XMLParser.getAttribute(el, XMLParser.CPU));
        String memory = XMLParser.getAttribute(el, XMLParser.MEMORY);
        String cluster = XMLParser.getAttribute(el, XMLParser.CLUSTER);

        ...

        SlurmExecutorInfo info = new SlurmExecutorInfo(...);
        if(di.getColor() != null)
            info.setColor(di.getColor());
        return info;
    }

    @Override
    public String getXSDDefinition() {
        return XSD_DEF;
    }

    @Override
    public void runAdditionalTestsOnElement(String name) {}
}
```

## 2.4 Implement the functionality of the executor

The final step before the new executor can be used in a workflow is to implement the actual functionality of the executor. This requires implementing the *SlurmExecutor* and *SlurmMonitorThread* classes used in the implementation of the *SlurmExecutorInfo* (see section 2.2) and *SlurmExecutorInfoParser* (see section 2.3) classes. In both cases, we again extend existing abstract classes, i.e. *ExternalScheduledExecutor* and *ExternalScheduledMonitorThread*.

The implementation of the *SlurmExecutor* and *ExternalScheduledMonitorThread* classes is very simple (see below). Here, the central function is the getExternalWorkloadManagerConnector function (lines 30-31), which creates an instance of the *SlurmWorkloadManagerConnector* class. The *SlurmWorkloadManagerConnector* class implements the functionality for interacting with the particular instance of Slurm running on a supercomputer or computing cluster. It again extends an abstract class *ExternalWorkloadManagerConnector* and implements the required functions for submitting, holding, releasing and canceling jobs and for getting information on execution of jobs. Since Slurm does not provide Java bindings, the implementation of these functions call external binaries using the Java Runtime.getRuntime function. For sake of brevity, the implementation of the *SlurmWorkloadManagerConnector* is not shown here but can be found in the *Watchdog* source code.

```
1  public class SlurmExecutor extends ExternalScheduledExecutor<
       SlurmExecutorInfo> {
2
3      private static ExternalScheduledMonitorThread<
           ExternalScheduledExecutor<?>> monitor;
4
5      public SlurmExecutor(Task task, SyncronizedLineWriter log, int
           retryCount, SlurmExecutorInfo execInfo) {
6          super(task, log, retryCount, execInfo);
7      }
8
9      public static void setExternalScheduledMonitorThread(
           ExternalScheduledMonitorThread<ExternalScheduledExecutor<?>>
           thread) {
10         monitor = thread;
11     }
12
13     @Override
14     public ExternalScheduledMonitorThread<ExternalScheduledExecutor<?>>
           getMonitor() {
15         return monitor;
16     }
17 }
18
19
20
21 public class SlurmMonitorThread extends ExternalScheduledMonitorThread<
       SlurmExecutor> {
22
23     private static ExternalScheduledMonitorThread<?> instance = null;
24
25     private  SlurmMonitorThread() {
26         super("SlurmMonitorThread");
27         this.connector = getExternalWorkloadManagerConnector();
28     }
29
30     protected static SlurmWorkloadManagerConnector
           getExternalWorkloadManagerConnector() {
31         return new SlurmWorkloadManagerConnector(new Logger());
32     }
33
34     public static void updateMonitorThread() {
```

```
            SlurmExecutor.setExternalScheduledMonitorThread((ExternalScheduledMonitorThread<ExternalScheduledExecutor<?>>) getMonitorThreadInstance());
    }

    public static ExternalScheduledMonitorThread<?> getMonitorThreadInstance() {
        if(instance == null || instance.isDead())
            instance = new SlurmMonitorThread();

        return instance;
    }
}
```

# 3 Using the new executor in a workflow

After implementing the required classes, including them in the *Watchdog* jar-file and copying the XSD file for the new element in the sub-directory of the *Watchdog* installation directory indicated in the implementation of the XML parser (see 2.3), the new executor can be used within a workflow like any other executor. An example for using the Slurm executor to run several sleep tasks is shown below.

```
<?xml version="1.0" encoding="UTF-8"?>
<watchdog xmlns:xsi="http://www.w3.org/2001/XMLSchema-instance" xsi:noNamespaceSchemaLocation="watchdog.xsd" watchdogBase="...">

    <settings>
    <processBlock>
        <processSequence name="loop" start="1" end="5" step="1"/>
    </processBlock>
    <executors>
        <slurm name="newExecutor" maxRunning="2" memory="250M" cpu="1" timelimit="0-0:15"/>
    </executors>
    </settings>

    <tasks>
        <sleepTask id="1" name="loop" executor="newExecutor" processBlock="loop">
            <parameter>
                <wait>[]s</wait>
            </parameter>
        </sleepTask>
    </tasks>
</watchdog>
```
