# Supplementary material for: Watchdog – a workflow management system for the distributed analysis of large-scale experimental data
Source: BMC Bioinformatics. 2018 Mar 13;19:97. doi: 10.1186/s12859-018-2107-4 (PMC5850912; doi:10.1186/s12859-018-2107-4)
Supplement: Supplementary file 4 — Computational overhead of Watchdog. Contains an analysis of the computational overhead of Watchdog and Snakemake for executing a workflow with a variable number of samples. (PDF 157 kb) [file 12859_2018_2107_MOESM4_ESM.pdf]

Watchdog – A Workflow Management System for the Distributed Analysis of Large-scale Experimental Data

Michael Kluge and Caroline C. Friedel

Additional File 4: Computational overhead of Watchdog

To estimate the computational overhead of running either Watchdog or Snakemake, we implemented a simple workflow consisting of five tasks (for Watchdog see Fig. 1) or rules (for Snakemake see Fig. 2). Each task or rule is dependent on the previous one and applied to a variable number of samples using either process blocks in Watchdog or wildcards in Snakemake. This simulates a next-generation sequencing analysis on numerous samples or replicates, consisting for instance of decompression of fastq.gz files (task/rule 1), quality control using FastQC (task/rule 2), read mapping (task/rule 3), conversion of SAM-files to BAM-files (task/rule 4) and indexing of BAM-files (task/rule 5). Due to the significant runtime of these analyses steps, which do not affect the computational overhead of the workflow management system used, we replaced each task by a 1s sleep command and a Linux touch command to create output files (necessary to implement dependencies in Snakemake). Files were written to and read from Linux shared memory (*/dev/shm/*) to exclude delays from accessing the file system.

Both workflows were run five times on the same computer (8 cores, Intel i7-3770, 3.40GHz, 32GB RAM, openSUSE Linux). For Watchdog, a local executor was used that allowed only one simultaneously running (sub)task. For Snakemake, one core was used and each rule allowed only one thread. Runtime was calculated using the Linux time command. Computational overhead of each workflow management system was calculated by subtracting the total time for the executed sleep tasks (= 1s $\times$ the number of tasks $\times$ the number of subtasks per task = 5s $\times$ number of subtasks per task) from the total runtime (see Fig. 3a). Furthermore, the computational overhead per executed subtask was obtained by dividing the total computational overhead by the total number of executed (sub)tasks (= number of tasks $\times$ the number of subtasks per task, see Fig. 3b). Results between the five repeats were highly reproducible and showed only little variation (Fig. 3).

This benchmark showed for both workflow management systems an overhead that was approximately linear in the number of tasks with a small offset for starting workflow execution. For Watchdog, this initial offset was slightly larger than for Snakemake ($\sim$4s compared to $\sim$1s, estimated with linear regression). In contrast, the overhead per executed (sub)task was lower for Watchdog than for Snakemake ($\sim$0.022s compared to $\sim$0.056s). As a consequence, with increasing number of subtasks, i.e. increasing number of samples or replicates, the difference between the computational overhead of Snakemake and Watchdog increased approximately linearly (see Fig. 3a). Nevertheless, compared to the runtime of the executed tasks, this difference was small. Thus, in total runtime, the differences between Snakemake and Watchdog were hardly noticeable (Fig 3c). Since real-life applications require tasks that take substantially longer than 1s to execute, we can conclude that the computational overhead of using either Watchdog or Snakemake is negligible. This likely applies to any commonly used workflow management system.

```xml
<?xml version="1.0" encoding="UTF-8"?>
<watchdog xmlns:xsi="http://www.w3.org/2001/XMLSchema-instance"
    xsi:noNamespaceSchemaLocation="watchdog.xsd" watchdogBase="...">
  <settings>
    <executors>
         <local name="local" default="true" maxRunning="1"/>
    </executors>
    <processBlock>
         <processFolder name="folder" folder="/dev/shm/samples/" pattern="*.txt" />
    </processBlock>
  </settings>

  <tasks mail="dummy@mail.com">
    <sleepTouchTask id="1" name="sleep1" processBlock="folder">
     <parameter>
         <wait>1</wait>
     </parameter>
    </sleepTouchTask>

    <sleepTouchTask id="2" name="sleep2" processBlock="folder">
     <dependencies>
         <depends separate="true">1</depends>
     </dependencies>
     <parameter>
         <wait>1</wait>
     </parameter>
    </sleepTouchTask>

    <sleepTouchTask id="3" name="sleep3" processBlock="folder">
     <dependencies>
         <depends separate="true">2</depends>
     </dependencies>
     <parameter>
         <wait>1</wait>
     </parameter>
    </sleepTouchTask>

    <sleepTouchTask id="4" name="sleep4" processBlock="folder">
     <dependencies>
         <depends separate="true">3</depends>
     </dependencies>
     <parameter>
         <wait>1</wait>
     </parameter>
    </sleepTouchTask>

    <sleepTouchTask id="5" name="sleep5" processBlock="folder">
     <dependencies>
         <depends separate="true">4</depends>
     </dependencies>
     <parameter>
         <wait>1</wait>
     </parameter>
    </sleepTouchTask>
  </tasks>
</watchdog>
```

Figure 1: *Watchdog* workflow for runtime evaluation. This workflow consists of 5 tasks with subtask dependencies between subsequent tasks. Subtasks for each task are created using a process folder block on the directory */dev/shm/samples/* in the Linux shared memory. A variable number of files in this directory were created before running the workflow to simulate different numbers of samples or replicates. A *sleepTouchTask* first sleeps for a specified number of seconds (in this case 1s) and then creates an empty file using the Linux *touch* command (as performed in the Snakemake workflow in Fig. 2). All tasks were executed on one core of a local executor, with at most one (sub)task running at the same time.

```
(SAMPLES,) = glob_wildcards("/dev/shm/samples/{sample}.txt")

rule all:
    input:  expand("/dev/shm/samples/{sample}_tmp5.txt", sample=SAMPLES)

rule rule1:
    input:  "/dev/shm/samples/{sample}.txt"
    output: "/dev/shm/samples/{sample}_tmp.txt"
    threads: 1
    shell:  "sleep 1; touch {output};"

rule rule2:
    input:  "/dev/shm/samples/{sample}_tmp.txt"
    output: "/dev/shm/samples/{sample}_tmp2.txt"
    threads: 1
    shell:  "sleep 1; touch {output};"

rule rule3:
    input:  "/dev/shm/samples/{sample}_tmp2.txt"
    output: "/dev/shm/samples/{sample}_tmp3.txt"
    threads: 1
    shell:  "sleep 1; touch {output};"

rule rule4:
    input:  "/dev/shm/samples/{sample}_tmp3.txt"
    output: "/dev/shm/samples/{sample}_tmp4.txt"
    threads: 1
    shell:  "sleep 1; touch {output};"

rule rule5:
    input:  "/dev/shm/samples/{sample}_tmp4.txt"
    output: "/dev/shm/samples/{sample}_tmp5.txt"
    threads: 1
    shell:  "sleep 1; touch {output};"
```

Figure 2: Snakemake workflow for runtime evaluation. The same workflow as for Watchdog (see Fig. 1) was implemented in Snakemake with 5 rules, each of which depends on the previous rule. Each rule consists of 1s sleep commands and a Linux *touch* command to create the corresponding output files for dependencies. A variable number of files in the */dev/shm/samples/* directory were created before running the workflow to simulate different numbers of samples or replicates. The workflow was executed on the same machine as the Watchdog workflow described in Fig. 1 using only one core and one thread per rule to make it comparable to Watchdog execution, which allowed only one running (sub)task at the same time.

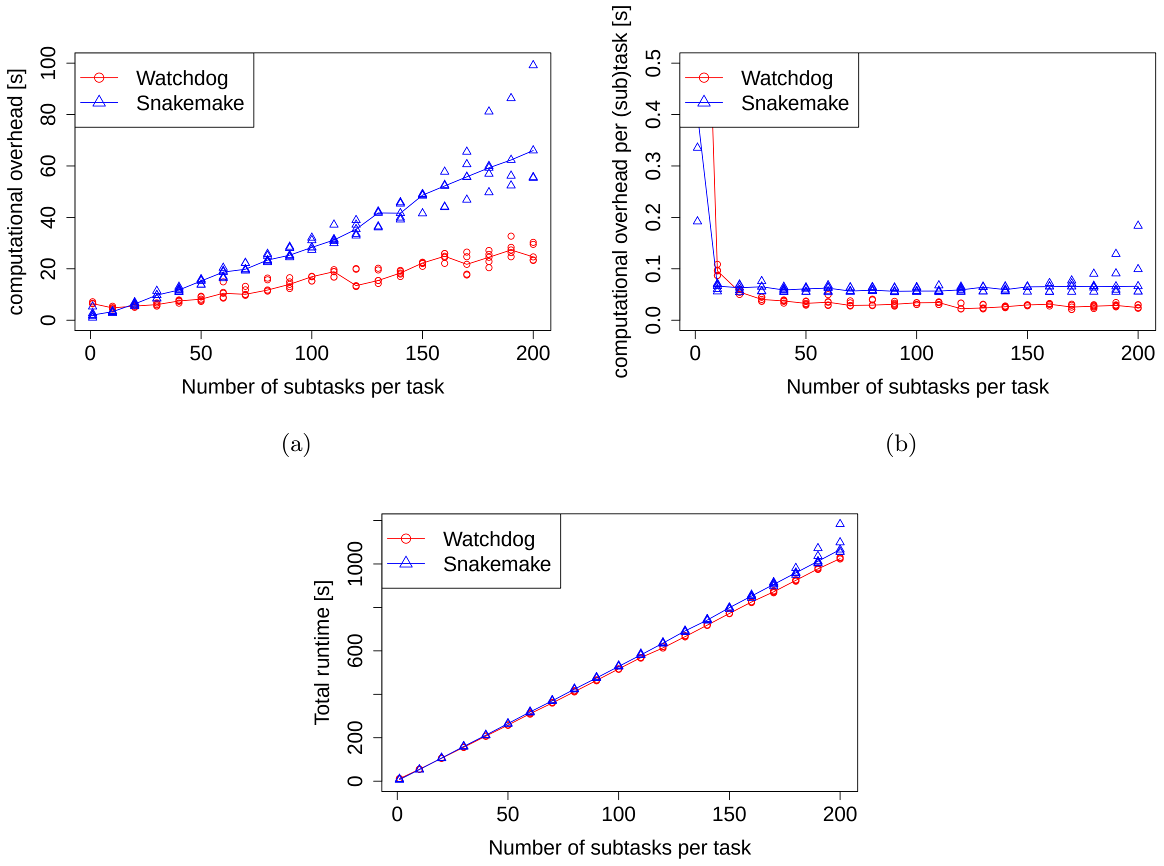

(a) (b)

(c)

Figure 3: Computational overhead for the Watchdog and Snakemake workflows described in Figs. 1 and 2. Results for five independent runs of each workflow are shown (circles for Watchdog and triangles for Snakemake) and median values are indicated by lines. In each case, the x-axis indicates the number of subtasks executed for each task or rule. Thus, the total number of sleep and touch commands is five times (for the five tasks/rules) the number indicated on the x-axis. Subfigure (a) shows the total computational overhead, which was calculated as total runtime minus the duration of all executed sleep tasks (= 5s × number of subtasks). (b) shows the additional runtime per executed (sub)task (calculated as computational overhead divided by 5 × number of subtasks per task). (c) shows the total runtime including the time for sleep and touch tasks.
